# Microbial acidification by N, S, Fe and Mn oxidation as a key mechanism for deterioration of subsea tunnel sprayed concrete

**DOI:** 10.1038/s41598-024-73911-w

**Published:** 2024-09-30

**Authors:** Sabina Karačić, Carolina Suarez, Per Hagelia, Frank Persson, Oskar Modin, Paula Dalcin Martins, Britt-Marie Wilén

**Affiliations:** 1 Department of Architecture and Civil Engineering, Chalmers University of Technology, Göteborg, 41296 Sweden; 2 Division of Water Resources Engineering, Faculty of Engineering LTH, Lund University, Lund, 221 00 Sweden; 3 Construction Division, The Norwegian Public Roads Administration, Oslo, 0030 Norway; 4 Department of Ecosystem and Landscape Dynamics, University of Amsterdam, Amsterdam, 1090 GE Netherlands; 5 Institute of Medical Microbiology, Immunology and Parasitology, Medical Faculty, Rheinische Friedrich-Wilhelms Universität, 53127 Bonn, Germany; 6 Müller-Sars Biological Station, Ørje, NO-1871, Norway; 7 Sweden Water Research AB, Lund, 222 35, Sweden; 8 Microbial Ecology Cluster, GELIFES, University of Groningen, Groningen, 9747 AG, Netherlands

**Keywords:** Fibre-reinforced sprayed concrete; subsea tunnels, Biodeterioration, Biofilm community, Amplicon sequencing, Metagenomics, Microbiology, Biogeochemistry, Ecology, Engineering, Materials science

## Abstract

The deterioration of fibre-reinforced sprayed concrete was studied in the Oslofjord subsea tunnel (Norway). At sites with intrusion of saline groundwater resulting in biofilm growth, the concrete exhibited significant concrete deterioration and steel fibre corrosion. Using amplicon sequencing and shotgun metagenomics, the microbial taxa and surveyed potential microbial mechanisms of concrete degradation at two sites over five years were identified. The concrete beneath the biofilm was investigated with polarised light microscopy, scanning electron microscopy and X-ray diffraction. The oxic environment in the tunnel favoured aerobic oxidation processes in nitrogen, sulfur and metal biogeochemical cycling as evidenced by large abundances of metagenome-assembled genomes (MAGs) with potential for oxidation of nitrogen, sulfur, manganese and iron, observed mild acidification of the concrete, and the presence of manganese- and iron oxides. These results suggest that autotrophic microbial populations involved in the cycling of several elements contributed to the corrosion of steel fibres and acidification causing concrete deterioration.

**Supplementary Information:**

The online version contains supplementary material available at 10.1038/s41598-024-73911-w.

## Introduction

Biofilms can have large economic impacts via, for example microbially induced corrosion^1^. Concrete biodeterioration, often referred to as microbially induced concrete corrosion (MICC), is a widespread problem causing damage to infrastructure such as pipelines, offshore infrastructure, sewer systems, and tunnels. In addition to the economic impact, this has significant environmental implications due to the large scale of this kind of structures. Concrete is the most common construction material globally, accounting for 8% of the global anthropogenic CO_2_ emissions^2^.

Water in contact with concrete can lead to deterioration of the cement hydrates and steel reinforcement by both abiotic and biotic processes^3–5^. External attack by aggressive waters on concrete and steel involves deterioration mechanisms such as calcium leaching, acid attack, sulphate attack, carbonation, magnesium attack and chloride attack^6–8^. Microorganisms, including bacteria, archaea, fungi, and algae, readily colonise surfaces immersed in water and form biofilms^9^. This provides them with access to nutrients and protection against predation, extreme environments, and aggressive compounds. Biofilm growth on concrete can result in acidifying processes during biogeochemical cycling, which promotes deterioration of concrete, where the cement paste matrix can be transformed and even dissolved^10–12^. Biofilms also catalyse corrosion of steel and other metals which are added to reinforce the concrete^13^.

In sewer systems, the main metabolic interactions leading to the deterioration of concrete involve sulfate-reducing bacteria (SRB) forming hydrogen sulfide when oxidising organic matter from sewage in anaerobic environments. Hydrogen sulfide is subsequently used as a substrate by sulfur-oxidising bacteria (SOB), decreasing pH and releasing sulfuric acid. This leads to partial dissolution of the cement paste matrix, gypsum formation and spalling^14–16^.

In marine environments, such as offshore infrastructures (e.g., gas and oil pipelines), less is known about metabolic interactions causing concrete degradation. The concrete is often reinforced with steel fibres, with the high alkalinity of the concrete initially protecting against corrosion^15^. In coastal infrastructure, sea walls, tunnels and other underground structures, sprayed concrete is often used for rock support^17^. Sprayed concrete is applied directly on the rock mass partly in contact with leaking water and forms a relatively thin layer with high surface roughness in comparison with cast concrete^18^. The establishment of biofilms on concrete surfaces can result in aggressive environments, altering the pH and producing corrosive metabolites in marine environments^4^. Sulfur reduction and oxidation can be important, especially in cases with oil-water systems found in off-shore structures^19^ possibly together with the oxidation of ammonia, iron, and manganese^15,18^.

In subsea tunnels, steel fibre-reinforced sprayed concrete is exposed to the intrusion of saline groundwater. Previous studies in several Norwegian subsea tunnels have shown a new form of biodeterioration of the steel fibre reinforced sprayed concrete, although the detailed mechanisms of the MICC are yet to be determined^18,20^. Biofilm with layered deposits of amorphous ferrihydrite (Fe-hydroxide) and manganese-oxides (buserite and todorokite) formed within a few weeks on the rough bioreceptive sprayed concrete surfaces in association with saline water leakages. This has been observed in the Oslofjord tunnel and several other Norwegian subsea tunnels^18^. The deterioration of the cement paste matrix involved leaching of calcium with increasing secondary porosity under the biofilm and apparent abiotic formation of non-cementitious minerals such as carbonates, magnesium-silicate hydrate (M-S-H) and thaumasite sulfate attack (TSA) at variable expense of the cement paste matrix deeper inside the concrete. Redox reactions within the biofilm resulted in mild acidification of biofilm water, which together with chloride contributed to depassivation of the steel fibre reinforcement, The biofilm caused oxidation of steel fibres and aqueous Fe and Mn, and formation of sulfuric acid, carbonic acid, organic acids and possibly also ammonia oxidation. Reduction of iron hydroxide, manganese oxides and sulfate also occurred depending on local conditions, envisaging a dynamic system with an impact on the acid producing reaction within the biofilms. The overall composite attack resulted in a weaking and thinning of the sprayed concrete and outer material loss at rates ranging from < 0.5 to 10 mm per year, and in an extreme case at a specific site > 100 mm within less than five years^18,20^.

Initial microbial surveys of the Oslofjord tunnel biofilms have revealed complex biofilm communities, which differed in structure between sites^21^. The community composition had a strong marine signature, with several microorganisms in the tunnel biofilms likely originating from the fjord sediments above the tunnel^22^. Iron-oxidising *Zetaproteobacteria* and ammonia-oxidizing bacteria and archaea typically found in seawater were frequently observed, and several novel taxa were detected^21–23^. However, the identity and function of key biofilm community members in relation to the observed MICC have not been established. For instance, despite the previously described widespread presence of Mn-oxides and evidence for sulfur transformations, manganese-oxidizing bacteria and sulfur-cycling bacteria are yet to be identified.

In this study, we aimed to identify the key microbial taxa and potential mechanisms responsible for the MICC and biomineral formation. The study was conducted at two sites over five years. Amplicon sequencing and metagenomics were used to characterize the biofilm community structure and temporal dynamics and to identify microorganisms and mechanisms involved in Fe, Mn, N and S transformations. Concrete deterioration and the formation of biominerals were evaluated using polarised light microscopy, scanning electron microscopy (SEM) and X-ray diffraction (XRD). Finally, the significance of biofilm age and other site-specific characteristics regarding effects of biodeterioration of steel fibre reinforced sprayed concrete are discussed.

## Materials and methods

### Sampling

Three locations in the Oslofjord subsea tunnel were sampled: the Pump Station (subsites P1 and P2) and the Test Site (T). These sites represent characteristic features of relatively fast sprayed concrete deteriorations involving Mn-Fe biofilm. The sampling points were chosen on the basis of monitoring by the Norwegian Public Roads Administration (NPRA) since 2004. The Oslofjord Test Site (T) was established by NPRA for long term investigations of typical subsea concrete attack.

The sprayed concrete at sites P1 and P2 was placed in 1999 and made with steel fibre reinforcement, CEM I, and silica fume. At site T, the sprayed concrete was placed in 2010 and made with fly ash cement (CEM II/A-V 42.5R, e.g. with ~ 18% fly ash), silica fume with steel fibre and polypropylene fibre reinforcement. Aluminium-sulfate was used as a setting accelerator at all sites. The water/binder ratio (w/b) at sites P1-P2 and site T were 0.42 and 0.43, respectively. This corresponds to Durability Class M45 according to the European concrete standard CEN - EN 206 Concrete - Specification, performance, production and conformity. Biofilms had developed in leakage areas shortly after the spraying operations at each site, except for site P1 which remained dry from 1999 to 2013. Biofilm started to develop also at site P1 after that enhanced water flow was observed. The similar concrete permeabilities (w/b-ratios) and different concrete and biofilms ages at the three sites makes this an ideal case for studying the effects of MICD over time.

Sampling of biofilms was carried out as previously described^21^ from the P1 and P2 and T sites over the years 2015–2020. Biofilm samples were collected using metallic tubes, whilst thin biofilms were scraped off the concrete with a scalpel. The biofilm samples were snap-frozen in an ethanol - dry ice mixture immediately at sampling, kept in dry ice during transportation, and stored at -80 °C until nucleic acid extraction. Finally, in September 2020, P1, P2 and T were selected for establishing a cross section from outer biofilms and deteriorated sprayed concrete. Sampling involved (a) biofilm for DNA extraction, SEM and XRD, (b) small chips of outer degraded concrete for SEM and (c) core extraction of concrete for thin sectioning and concrete petrography by polarised microscopy and SEM.

### DNA extraction, amplification, and sequencing

During the investigation period, DNA was extracted from a total of 129 biofilm samples (Supplementary material, Table S8) using the Fast DNA spin kit for soil (MP Biomedicals) following the manufacturer’s protocol.

#### Amplicon sequencing

Amplicon sequencing of the 16 S rRNA gene was carried out following Karačić et al.^21^. Briefly, the V4 region was amplified, using the forward and reverse primers 515ʹF and 806R, respectively, to cover both bacteria and archaea^24,25^. PCR products were purified and pooled in equimolar amounts prior to sequencing on a MiSeq (Illumina) using the MiSeq Reagent Kit v3, generating 300 bp-long paired-end reads.

Reads were processed using the VSEARCH^26^ and DADA2^27^ pipelines. Based on the two count tables, a consensus table of amplicon sequence variants (ASVs) detected by both pipelines was generated using qdiv (https://github.com/omvatten/qdiv) according to Modin et al.^28^. Alpha and beta diversity were calculated using the Hill number framework^29,30^. The parameter q is the diversity order. At q = 0, all ASVs are considered equally important, while at q = 1, each ASV is weighted according to its relative abundance. For alpha diversity (^q^D), this means that ^0^D is equal to the richness (all ASVs in a sample) while ^1^D is interpreted as the number of “common” ASVs. The corresponding dissimilarity index (^q^d) shows the fraction of all (^0^d) and “common” (^1^d) ASVs not shared between a pair of samples^28,29^. Data visualization and alpha and beta diversity calculations were conducted within the qdiv pipeline. Changes in alpha and beta diversity over time were analysed using linear regression in scipy^31^.

#### Metagenomics

DNA extractions from eight samples, obtained from sites P2 and T from the years 2016, 2017, 2019, and 2020, were selected for shotgun metagenomics. Sampling was only done from site P2 since amplicon sequencing showed minor variation in the microbial community between P1 and P2. Sequencing, assembly and binning are described in detail elsewhere^22^. Binning was conducted with MetaBAT2 v2.15^32^ and BinSanity v0.5.3^33^, with dereplication using DASTool v1.1.2^34^. After assessing quality with CheckM^35^, only MAGs with less than 10% contamination and more than 50% completeness were chosen. The relative abundance of MAGs was estimated with coverM v0.6.1 (https://github.com/wwood/CoverM) with the *relative_abundance* parameter in *genome* mode. Taxonomic classification was carried out with GTDB-Tk v2.1.0^36^ using the GTDB R207_v2 reference^37,38^. Genomes were annotated with DRAM v1.0^39^. Genes linked to iron oxidation and iron reduction were identified using FeGenie^40^. Auxiliary dissimilatory sulfate reduction genes were identified with DiSCo^41^. Amino acid sequences for the DsrA tree were retrieved from MAG annotations and NCBI^42^. Sequences were aligned with muscle v3.8.31^43^, trimmed with trimal v1.4.rev22^44^ using the gappyout option, and used for tree building on FastTree v2.1.10^45^ using the Jones-Taylor-Thorton maximum-likelihood model. For MAGs classified as SBBL01, a phylogenetic tree using 92 genes was built with UBCG v3.0^46^. The tree was edited with iTOL^47^. Cyc2 was identified with FeGenie. Genes with homology to PCC genes were identified by BLASTP (bitscore > 300) to sequences retrieved from *Ca.* Manganitrophus noduliformans Mn-1^48,49^. The average amino acid identity (AAI) between SSBL01 genomes was estimated with the Kostas Lab tool (http://enveomics.ce.gatech.edu/g-matrix/index). For this, genomes were gene-called with Prodigal v2.6.3^50^, and amino acid fasta files were used as input. To assess the presence of microbial eukaryotes in the biofilm, Tiara v1.0.3 was used for classification of the contigs^51^.

### Water chemical analysis

Water samples were collected in sterile 500 ml bottles, from both the dripping water upstream of the sprayed concrete and from the water being exposed to the biofilm and concrete. The water samples were stored on dry ice during transportation to the laboratory. All water samples were filtered (0.45 μm) before analysis. Selected metals were quantified by quadrupole Inductivity Coupled Plasma-Mass Spectrometry (ICP-MS) using an iCAP Qc (Thermo Fischer Scientific) with FAST sample induction system. Prior to analysis the samples were acidified with 1% high purity HNO_3_. The instrument was operated in the standard and kinetic energy discrimination modes with He as a collision gas. Calibration was performed with multi-element standard solutions and an internal standard (In 10 ppb) was continuously injected during analysis. Dissolved ions were analysed using a Dionex ICS-900 ion chromatograph (Thermo Scientific). Samples were diluted with MilliQ ultrapure water to reach a conductivity < 200 µS cm^− 1^. Additional analyses of ions, DOC, pH and alkalinity were performed at a commercial laboratory (ALS Laboratory Group Norway AS). In addition, pH measurements were performed on site using a handheld pH-Eh meter and pH-strips.

### Analysis of concrete and biominerals

Concrete samples (chips and cores) and associated biofilms were first examined visually. Polished thin sections, prepared with a non-fluorescent blue dye using water as a coolant, were first examined under a standard polarising microscope. Selected domains of biofilm, concrete chips and thin sections were analysed by scanning electron microscopy (SEM) using a Hitachi S–3600 N. X-ray element maps and backscatter electron (BSE) images were captured and followed by spot analyses (spot size 5 μm). The instrument was equipped with a Bruker XFlash^®^ 5030 energy dispersive X-ray detector (EDX), running on Quantax 400 (Esprit 1.9), for semi-quantitative elemental analysis and hyperspectral mapping, with < 127 eV FWHM at MnKα energy resolution. Samples were analysed in variable pressure (VP) mode (20 Pa), 15.0 kV accelerating voltage and approximately 50 nA beam current. The spot size was 5 μm.

For X-ray diffraction (XRD), small samples were ground in agate mortars under ethanol and left to dry before being mounted with a few drops of ethanol on sample holders and run in a Siemens D 5005 Spectrometer. Diffractograms were recorded from 2^o^ to 60^o^ with step size 0.05^o^ on the 2-Theta scale for 58 min. The instrument was set to 40 kV and 40 nA using Ni-filtered CuKα radiation with wavelength λ = 1.54 Å. The diffractograms were checked versus the Powder Diffraction Files database from the International Centre for Diffraction Data (PDF4 + 2020 database).

## Results

### Water chemical composition reflected biodeterioration processes

The water leakages acting on sprayed concrete at the investigated sites in the Oslofjord tunnel are saline groundwater. The saline groundwater in the Oslofjord tunnel contained high concentrations of Cl^−^ (19–22 g l^− 1^), Na^+^ (9–10 g l^− 1^), SO_4_^2−^ (2.6–3.3 g l^− 1^), Mg^2+^ (0.8–2.2 g l ^−^1) and low concentrations of NO_3_^−^ (≤ 3 mg l^− 1^), NH_4_^+^ (< 0.3–1.2 mg l^− 1^), alkalinity (2.0-3.1 mmol l^− 1^) and DOC (< 1.5–3.6 mg l^− 1^). There was a variation in water chemistry over time within each site (Figure S1, Tables S1-S3). After exposure to the biofilm-covered concrete, Ca concentrations in water increased (paired T-test, *p* < 0.01), being compatible with the observed leaching of the cement paste (Figure S1, S3-S5). The Mg concentrations varied over time, which likely reflects variable interaction with the concrete material involving the formation of brucite (Mg(OH)_2_) and magnesium-silicate-hydrate (M-S-H) at the expense of the portlandite (Ca(OH)_2_) and calcium-silicate-hydrate (C-S-H) in the cement paste matrix. Likewise, variation in sulfate concentrations may reflect precipitation of gypsum and formation of thaumasite at the expense of the C-S-H (Figures S3-S6). Aqueous Fe was consistently low, whilst the Mn concentrations were generally higher after exposure to biofilms despite significant precipitation of Mn oxides (Figure S1-S3). In situ, pH measurements of biofilm waters were mildly acidic at low flow rates, but similar to ground water and seawater at times with high water flow (Figure S7). According to the European concrete standard EN 206, the ambient waters represent Exposure Class XA3, which may lead to deterioration also in the absence of biofilm unless the water/binder (w/b) ratio is 0.40 (Durability Class M40). The sprayed concretes at the investigated sites were made with w/b-ratios of 0.42–0.43 (Durability Class M45 with somewhat higher permeability) and must be regarded as slightly vulnerable in comparison with a standardised mix design. It should be noted that the standard environmental classification according to CEN – EN 206 does not account for the effects of biofilms^11,18^.

### Biodeterioration of sprayed concrete varied depending on biofilm age and pH-history

**Fig. 1:**
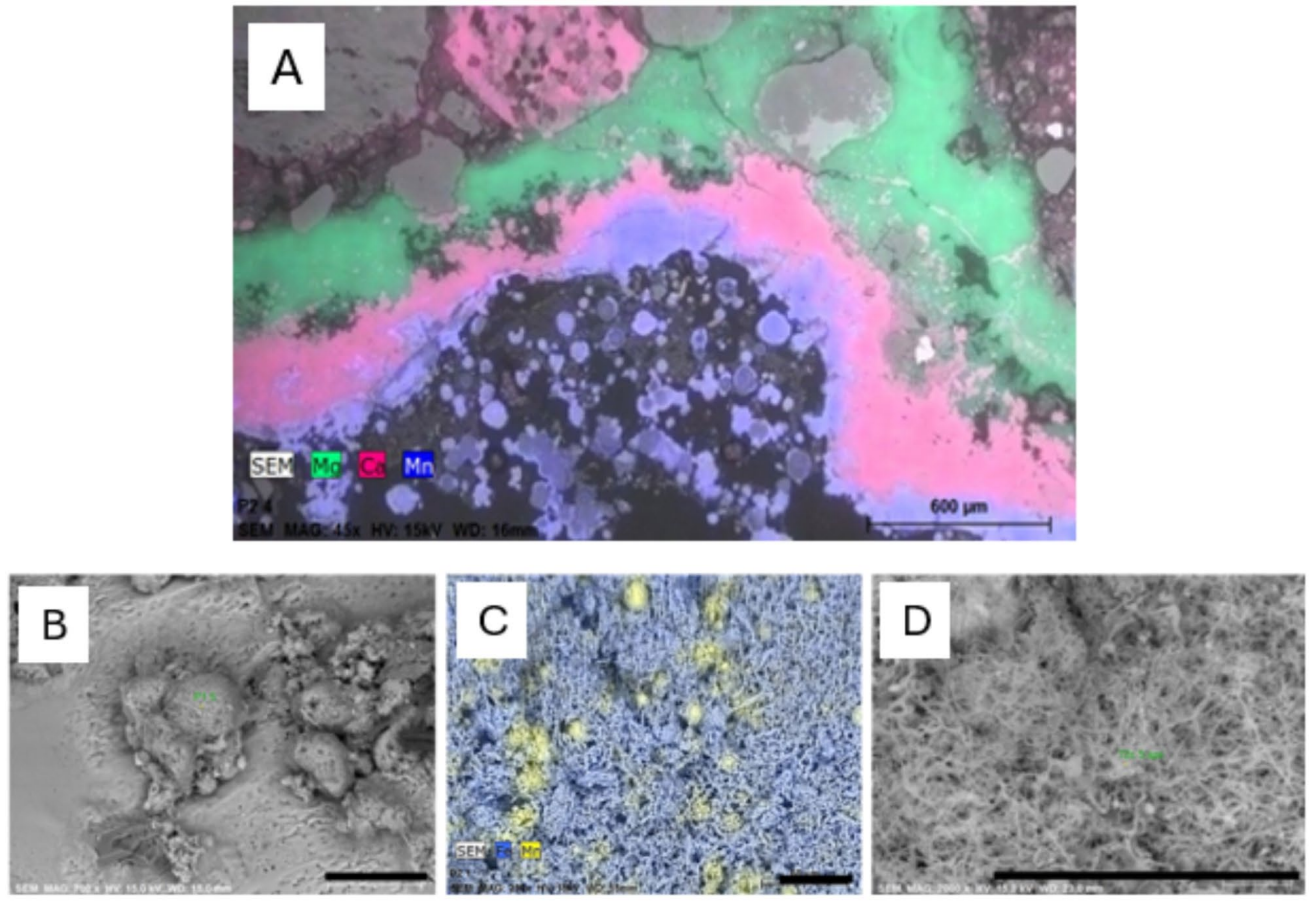
SEM and X-ray element map from thin section at location P2 (**A**). Mn-oxide (blue) formed on outermost sprayed concrete underneath slimy Fe-rich biofilm, (not preserved in the thin section, located at the lower part of the image). Notice successive layers of calcium carbonate (pink) derived from leaching of the cement paste and magnesium silicate hydrate (M-S-H) formation (green) at the expense of calcium silicate hydrate (C-S-H) further inside, scale bar = 600 μm; and selected SEM images of outer biofilms at site P1: Mn-rich biofilm (globules) on a substrate of pitted NaCl with minor presence of Fe-rich filaments (**B**); site P2: X-ray map of Mn-rich bacterial cells (light yellow) within Fe-rich stalked bacterial cells (blue), partly twisted (**C**); and site T: Fibrous Fe-rich stalks and smaller, round bacterial cells (**D**). Scale bars = 40 μm.

Water leakages were associated with the growth of biofilm on the sprayed concrete surfaces. SEM analysis of cross-sections of concrete cores showed that underneath a soft Fe-rich biofilm, Mn-oxides occurred frequently on concrete surfaces (Fig. 1A). Deterioration of the sprayed concrete was observed underneath these biofilms, and biofilms at the sites changed appearance over time (Figure S2).

Site P was divided into two different sub-sites (P1 and P2). The sprayed concrete at sites P1 and P2 is from 1999. Site T had concrete established in 2010. Biofilm emerged in connection with tunnel leakages soon after the establishment at sites P2 and T, whereas at site P1, biofilm started to emerge in 2013 after enhanced water flow.

At P1, the pH of water in contact with the biofilm was 6.65 in 2020 (Table S7), which was lower than the pH in the previous years (Figure S7) suggesting progressive onset of reactions in the biofilm. This was linked to lower water flow and a change in appearance towards darker biofilm colour. In 2020, the concrete underneath this biofilm appeared to be quite sound, likely reflecting the short period of decreased pH. The effect of biodeterioration in the form of friable concrete was marginal, reaching about 5 mm, without obvious signs of outer material loss. The friable part of the cement paste was Ca-leached and carbonated, with some influence of magnesium attack a bit deeper inside. Steel fibre corrosion was restricted to the concrete surface layers in direct contact with biofilm and the carbonated layers beneath (Tables S4, S5, Figure S3).

Site P2 was characterised by outer friable concrete and material loss beneath the biofilm. Here, concrete degradation has been observed underneath layered Mn-Fe biofilm for more than a decade prior to this study^18^, with decreased pH (5.5–7.2) in periods with slowly seeping water through the biofilm and higher pH (7.7–8.2) at periods of higher water flow (Figure S7). In 2020, pH was 6.85 (Table S7) and the biofilm was characterised by layered mineralised Mn and Fe (Fig. 1C and S4E). Thaumasite sulfate attack (TSA) with complete transformation of C-S-H occurred in a 10–15 mm layer immediately underneath. This contrasts with earlier results which rarely showed thaumasite in the surface region^18^, suggesting that the significant mass loss and enhanced porosity influenced by acidic water had paved the way for deeper diffusion of sulfate and bicarbonate and caused TSA concrete portions in regions with remaining alkaline pH. The thaumasite-rich layer was later extensively dissolved when reached by circumneutral to acidic saline water, causing a high secondary porosity. The cement paste further inside was leached and partly transformed into M-S-H with shrinkage cracks (Tables S4, S5, Figure S4). As for site P1, steel fibre corrosion was restricted to concrete in contact with biofilm and the leached outer concrete with carbonates in the presence of ambient chloride rich water.

At site T, water seeps were small resulting in layered, rusty brown and black biofilm with pH 6.2 to 7 in the period from 2010 to 2018. From 2018 to 2020, the water flow decreased further with surface drying of the biofilm (Figure S2A, D) and pH about 7.2 (Table S7). The outer 5 mm of the cement paste matrix was friable and leached with formation of popcorn calcite, M-S-H and brucite. Further inside, leaching and formation of M-S-H were observed, whilst thaumasite and calcite had formed near the contact zone with rock mass (Table S5, Figure S5). Steel fibre corrosion was limited, as for the other sites (above).

The biofilms at all three sites consisted of Mn-rich deposits and outer Fe-rich fibrous filaments and Fe-rich globules and local gypsum (Fig. 1B-D, Figure S6). XRD-analysis showed that the Mn-oxides buserite and todorokite, as well as amorphous ferrihydrite, formed as biominerals (Table S6). Next to oxygen, Mn-oxides are some of the strongest oxidising agents in the environment^52^, being stable at Eh about 0.4 to 0.5 V at the present pH-conditions. However, iron sulfide marcasite was detected in the biofilm at P2 (Table S6). Marcasite was also found at P2 in soft biofilm in 2005, reflecting sulfate reduction. Redox conditions within the biofilms are complex, involving great variation at small scales^18^. The final pH and Eh conditions in bulk waters running from biofilm are reported in Table S7.

**Fig. 2:**
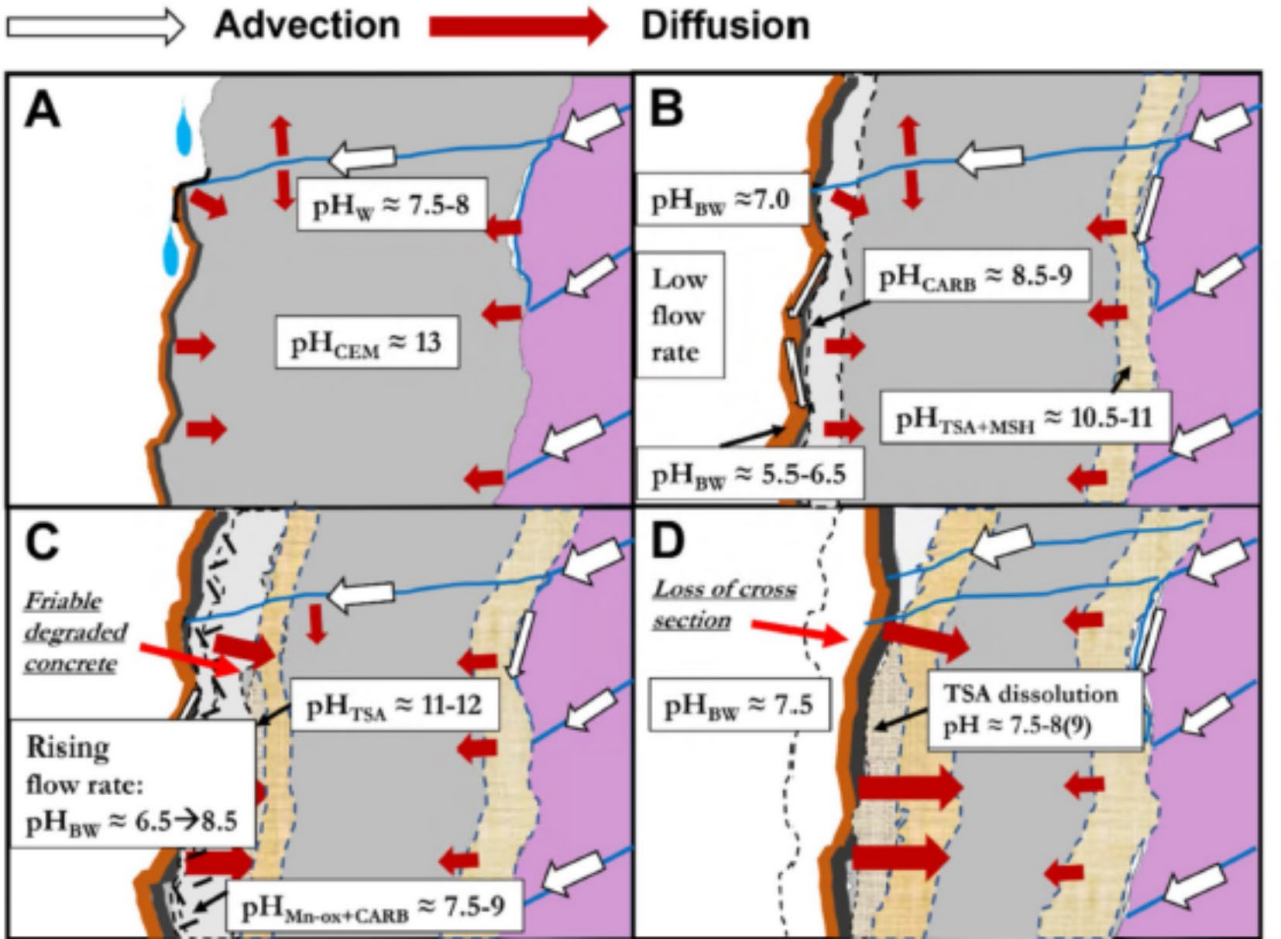
Schematic development of the concrete degradation. (**A**) initial biofilm formation, (**B**) degradation at a low flow rate, (**C**) continued degradation at an elevated flow rate, (**D**) effects of long-term degradation. The cross section shows sprayed concrete (grey) in contact with jointed rock mass (pink) with the development of deterioration underneath the biofilm (brown and black). The reaction zones with characteristic pore fluid pH and inward increasing pH gradient are indicated (light grey, beige). Friable degraded concrete contains carbonates and Mn-oxide (grey stippled). The different pH values in the reaction zones are based on the stability of minerals^18^, See text for an explanation. *W* water, *CEM* cement, *CARB* carbonate, *TSA* thaumasite sulfate attack, *Mn-ox* manganese oxide, *BW * biofilm water.

Proposed mechanisms of development of sprayed concrete deterioration are illustrated in Fig. 2. At an early stage, biofilm can form from a single water leakage (Fig. 2A). At a low flow rate, acidification reactions take place within the biofilm, leading to biodeterioration involving Ca-leaching, formation of secondary non-cementing minerals (e.g., M-S.H and thaumasite) increasing porosity (Fig. 2B). In the event of an increased flow rate, the saline groundwater with mildly alkaline pH will predominate over acid producing reactions within the biofilm (Fig. 2C). If the site already has suffered acidification for some few years, the effect of biodeterioration will continue due to enhanced secondary porosity after the leaching of the concrete. Thaumasite underneath the biofilm will dissolve at pH < 10–11 and increase the porosity even further, similar to what was observed at site P2 (Fig. 2D). Eventual later stage acidification will further accentuate the attack. However, in cases where the pH in biofilm water has always been in the absence of acids, e.g., at high flow rates, similar to the situation at site P1, the concrete will be less influenced by deterioration.

### Biofilm communities became more diverse over time

**Fig. 3:**
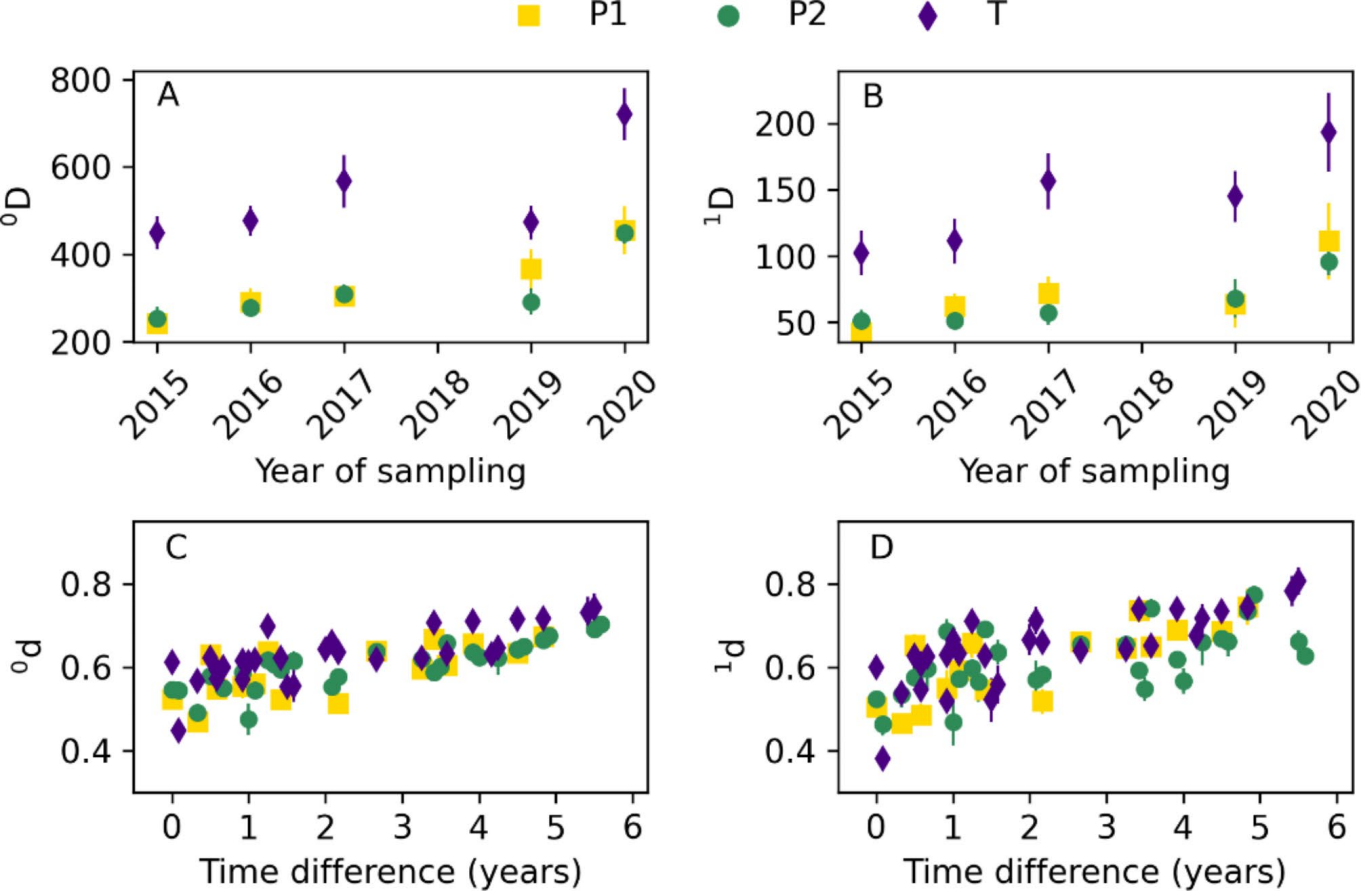
Alpha diversity of biofilm communities over time (**A**,**B**) and dissimilarity between biofilm communities were analysed at different time points (**C**,**D**). Both alpha diversity and dissimilarities were analysed using Hill-based indices with a diversity order of 0 (**A**,**C**) and 1 (**B**,**D**). Average values for each year and time difference are shown. The error bars represent the standard error of the mean.

Using amplicon sequencing of the 16 S rRNA gene (16 S), 129 biofilm samples collected from 2015 to 2020 were analysed. Alpha diversity was estimated as Hill numbers, at the diversity order of 0 (^0^D), equivalent to richness, and 1 (^1^D), taking the relative abundances of the ASVs into account. Alpha diversity was higher at site T than P for both ^0^D and ^1^D (Welch t-test, *p* < 0.001), but no difference was observed between sub-sites P1 and P2 (Welch t-test, *p* > 0.05). At both sites, alpha diversity increased over time for both diversity orders, with particularly high values observed in the biofilm samples taken in September 2020 (Fig. 3A, B). This increase over time was statistically significant (*p* < 0.05) for all locations and diversity orders except site T and diversity order 0.

To unravel potential differences in microbial communities between sites and over time, beta diversity was estimated. The P and T microbial communities differed (PERMANOVA, *p* < 0.001, Figure S9), as did communities from sub-sites P1 and P2 (PERMANOVA, *p* < 0.05). To investigate community changes over time, dissimilarity values were plotted at increasing time differences (3C, D). Longer time differences between the two samples correlated with higher dissimilarity, indicating a slow and gradual succession of the biofilm communities (Fig. 3C, D). Increases in dissimilarity over time were statistically significant (*p* < 0.001) for all locations and diversity orders.

### Novel marine microbial taxa in the Oslofjord subsea tunnel

**Fig. 4:**
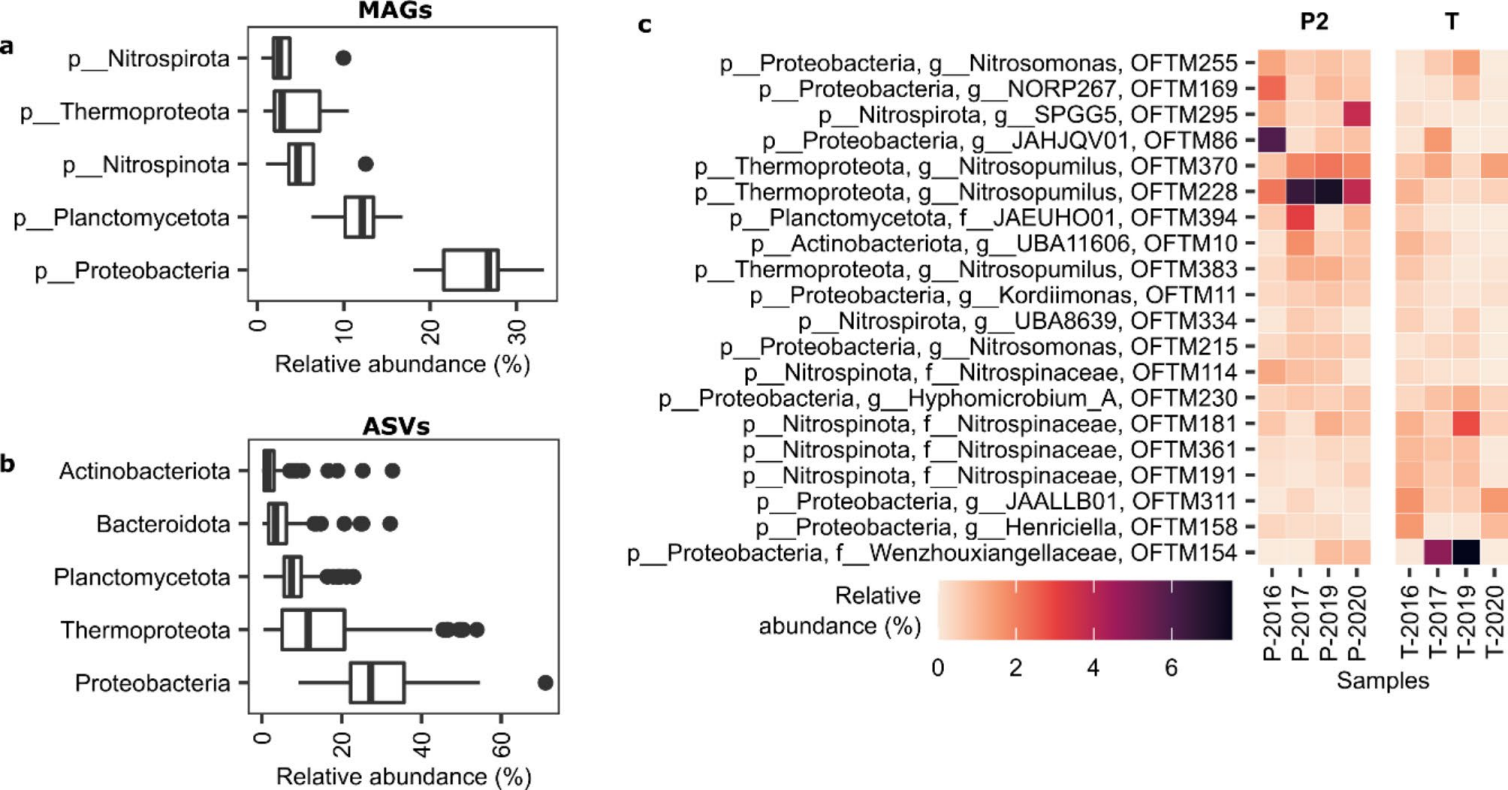
Relative abundance of taxa in Oslofjord tunnel biofilms: top five phyla among MAGs (**A**); top five phyla among ASVs (**B**); top 20 MAGs by median relative abundance (**C**). The boxplots (**A**,**B**) show median (vertical bold line), 25th and 75th percentiles (box delimiters), minimum and maximum values excluding outliers (horizontal lines), and outliers (dots) with *n* = 8 (**A**) and *n* = 129 (**B**).

By using shotgun metagenomics, 401 MAGs^22^ were reconstructed from sites T and P2 over the years 2015–2020, of which 3 belonged to *Archaea* and 398 belonged to *Bacteria*. Only 0.55% the sequence assembly were contigs classified as eukaryotic and no draft genomes of microbial eukaryotes could be reconstructed. In both the 16 S and metagenome datasets, biofilm communities were dominated by taxa in the phylum *Proteobacteria*, (*Pseudomonadota*) with an average relative abundance of 25.6% in the 16 S dataset and 37.2% among the MAGs. *Thermoproteota* (*Archaea*), *Planctomycetota*,* Bacteroidota* and *Actinobacteriota* (*Actinomycetota*) were thereafter the second to fifth most abundant phyla among the 16 S-based amplicon sequence variants (ASVs), while among MAGs these were *Planctomycetota*,* Nitrospinota*,* Thermoproteota* and *Nitrospirota* (Fig. 4B,C).

In the 16 S dataset, 79% and 60% of ASVs could not be classified at genus and family level, respectively. Similarly, of the 401 MAGs, only 186 could be classified at the genus level using the GTDB taxonomy. Among the known genera, many were of marine origin, such as *Nitrosopumilus*, *Nitrospina*, *Robiginitomaculum* and *Marinicaulis*. Adaptations to osmotic stress were also common, with 36 MAGs having an ectoine synthase-encoding gene (*ectC*), and thus the potential to produce the osmoprotectant ectoine^53^. Forty-one MAGs had the potential to produce hydroxyectoine via ectoine hydroxylase (*etcD*)^53^, while 33 MAGs had a gene encoding a betaine aldehyde dehydrogenase (*betB*), for production of the osmoprotectant glycine betaine^54^.

### Sulfur oxidation potential was widespread, but sulfur reduction potential was limited across biofilm community members

**Fig. 5:**
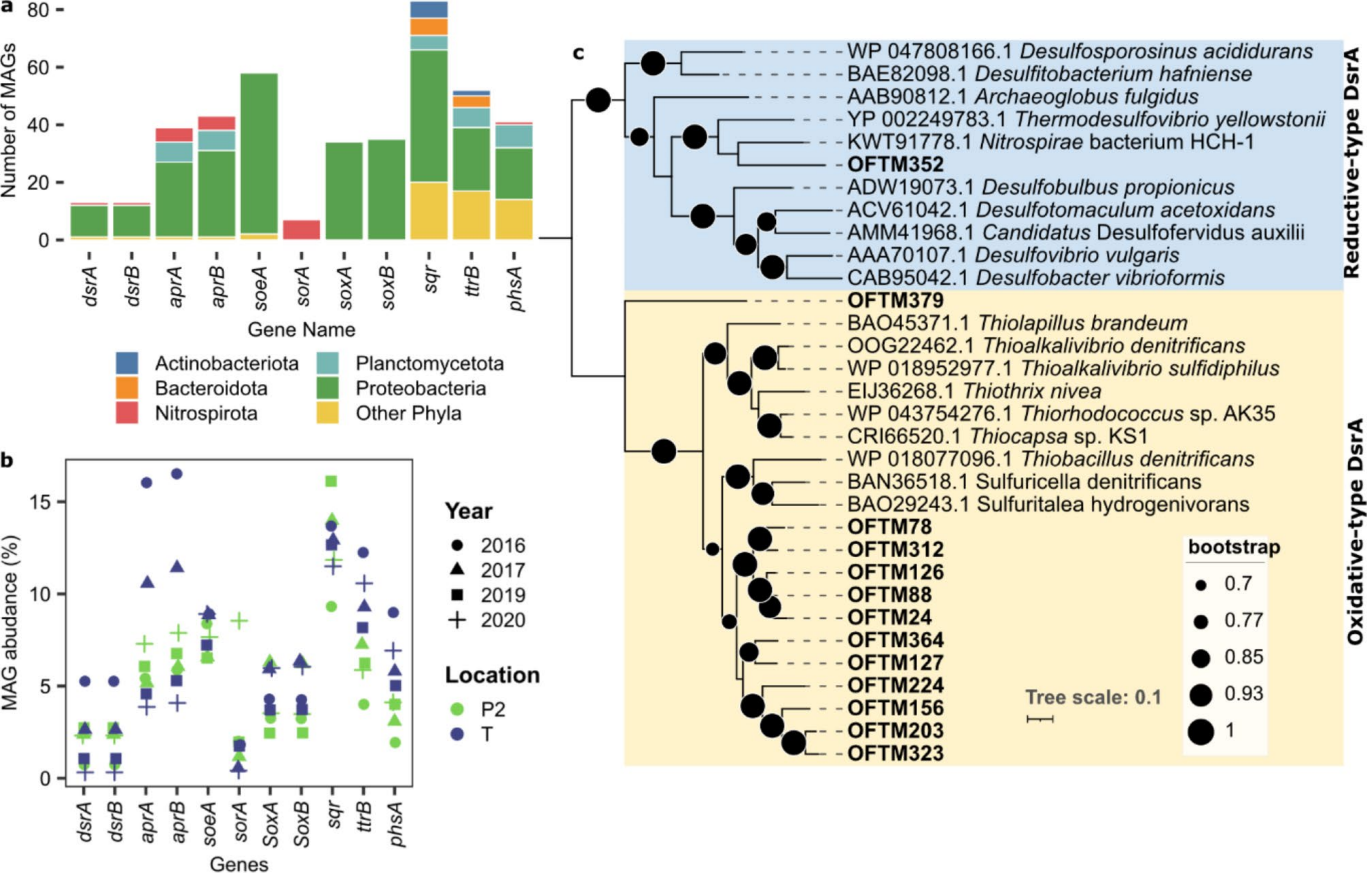
Genes encoding proteins for sulfur reduction or oxidation: numbers of MAGs that had these genes (**A**); relative abundances of MAGs that had these genes (**B**); phylogenetic tree of DsrA sequences retrieved from MAGs in this study (in bold) as well as reference genomes (NCBI accession numbers are provided) (**C**).

Genes involved in sulfur cycling and metal oxidation in MAGs were searched for, given that these processes have been previously demonstrated to be involved in MICC at the Oslofjord tunnel^18^. The genes adenylyl-sulfate reductase (*aprAB*) and dissimilatory sulfite reductase (*dsrAB*) encode proteins commonly linked to dissimilatory sulfate reduction, where AprAB together with sulfate adenylyltransferase (Sat) reduces sulfate to sulfite, followed by reduction of sulfite to sulfide by the DsrABC complex. These genes were identified in 13 MAGs (Fig. 5A, S10) mainly affiliated to *Proteobacteria*, but also in one *Nitrospirota* MAG and one *Nitrospinota* MAG. The relative abundance of these MAGs ranged between 0.3% and 4.2% (Fig. 5B). However, in some bacteria, the dsr pathway operates in the reverse direction and it is used for sulfur oxidation^55,56^. In 12 MAGs, *dsrA* clustered with oxidative-type reference sequences, while in one MAG, affiliated to the *Thermodesulfovibrionia* class, a reductive-type *dsrA* was identified (Fig. 5C, S10). This MAG had an average relative abundance of 0.06%.

The dominance of sulfur oxidation in the dsr pathway is also supported by an analysis of the dsr auxiliary genes (Figure S10), which revealed in these MAGs the presence of d*srEFH* genes typically associated with sulfur oxidation, while the dsrD gene, commonly linked to sulfur reduction^57^ was absent. Genes for thiosulfate oxidation via the SOX pathway (*SoxABCXYZ*) were identified in 36 MAGs affiliated to *Proteobacteria* (Fig. 5A, Figure S10), ranging from 2.5 to 6.3% relative abundance (Fig. 5B). In addition, the *soeA* and *sorA* genes for oxidation of sulfite to sulfate^58^ where present in 58 and 7 MAGs, respectively (Fig. 5A), where MAGs with *soeA* ranged from 6.5 to 8.9% and MAGs with *sorA* from 0.41 to 8.5% (Fig. 5B). The potential of sulfide oxidation to polysulfide was high, as evidence by the presence of sulfide: quinone oxidoreductase (sqr), identified in 83 MAGs, that summed between 9.3 and 16.1% (Fig. 5A,B).

Alternative respiratory sulfur reduction pathways include tetrathionate reduction. The tetrathionate reductase-encoding gene (*ttrB*), for the reduction of tetrathionate to thiosulfate, was present in 52 MAGs ranging from 4.0 to 12.2% (Fig. 5A,B). Thiosulfate reductase-encoding genes (*phsA*), linked to the disproportionation of thiosulfate into hydrogen sulfide and sulfite, were identified in 41 MAGs ranging from 1.9 to 9.0%. (Fig. 5A,B). The abundance of MAGs with *ttrB* and *phsA* genes was higher in location T than in location P2 (Fig. 5A,B).

### Potential for iron oxidation and reduction was widespread among the biofilm community members

**Fig. 6:**
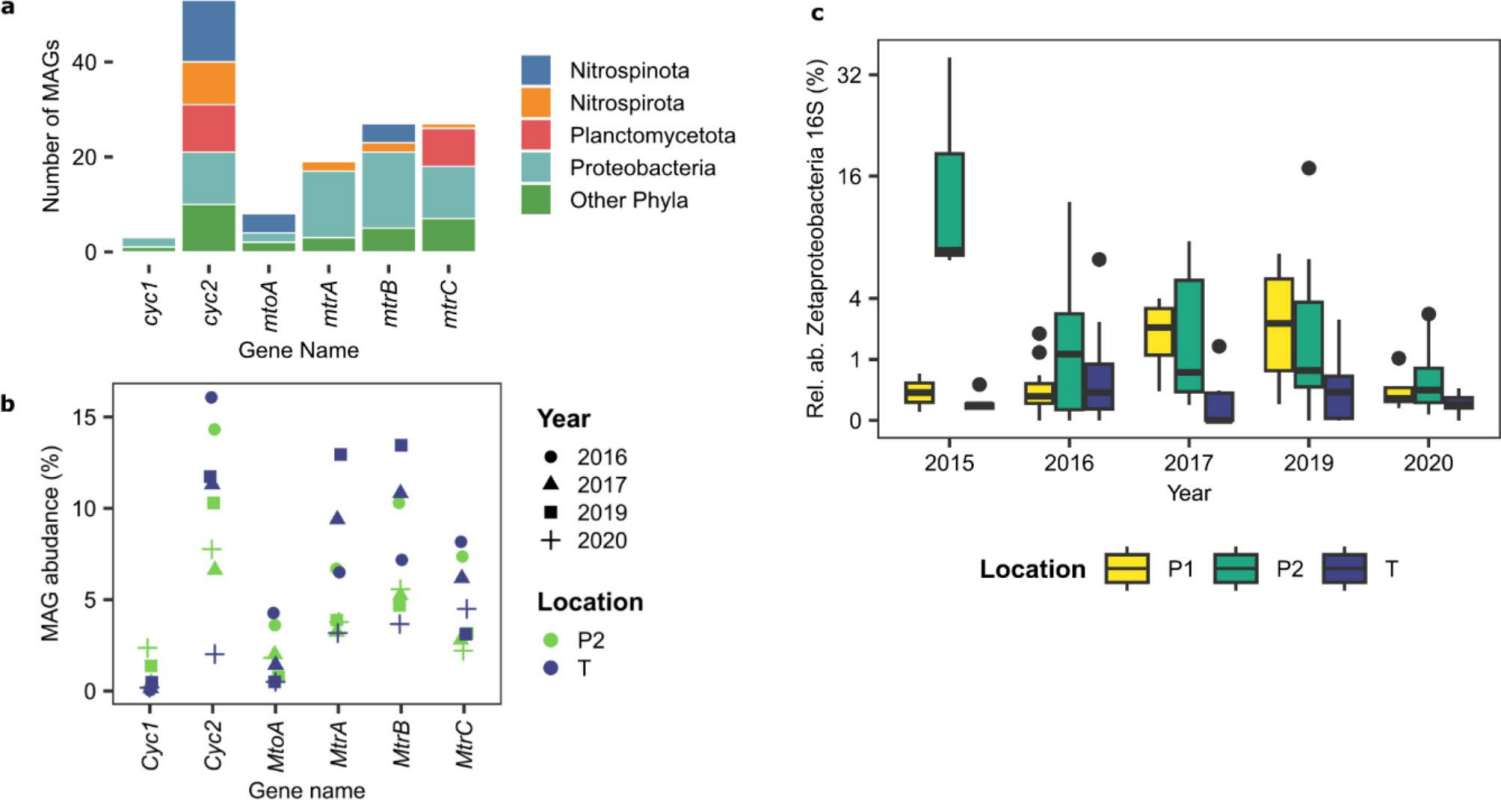
Potential for iron oxidation and reduction in microbial community members of Oslofjord tunnel biofilms. Number of MAGs with genes for iron oxidation and reduction (**A**); relative abundance of MAGs with genes for iron oxidation and reduction (**B**); and relative abundance of *Zetaproteobacteria* in the 16 S dataset showing median (horizontal bold line), 25th and 75th percentiles (box delimiters), minimum and maximum values excluding outliers (vertical lines), and outliers (dots).

The relative abundance of MAGs with potential for iron oxidation ranged between 5% and 20%. A gene encoding the cytochrome Cyc2 protein, which is an iron oxidase common among neutrophilic iron oxidisers^59,60^, was present in 51 out of 401 MAGs (Fig. 6B). Five of them were classified as *Zetaproteobacteria*, a group of marine iron-oxidising bacteria^59^. *Zetaproteobacterial* MAGs had a relative abundance varying between zero and 2.2% in biofilms. In 16 S rRNA gene datasets, *Zetaproteobacterial* relative abundance varied between zero and 35%, with an average of 1.7%, and higher abundance in site P relative to T (DESeq2, p_adj_ < 0.01) (Fig. 6C). Other MAGs containing a Cyc2-enconding gene were affiliated to *Proteobacteria* (4), *Nitrospirota* (15), *Nitrospinota* (8), and *Planctomycetota* (12) (Fig. 6A). A gene encoding the multiheme *c*-type cytochrome MtoA has also been linked to iron oxidation^61^ and was found in eight MAGs. These were affiliated to *Nitrospinota* (4), *Proteobacteria* (2), *Acidobacteriota* (1) and *Myxococcota_A* (1) MAGs (Fig. 6A, B).

The potential to reduce iron was identified in 34 MAGs that summed between 4% and 11% in relative abundance. Genes encoding MtrABC protein complexes, which in *Shewanella* have been linked to iron and manganese reduction^62^ were found in nine MAGs. Five of them were classified in the family *Woeseiaceae*, a group of bacteria that are commonly observed in marine sediments^63^.

### Potential for autotrophic manganese oxidation was identified in *Nitrospirota* genomes

**Fig. 7:**
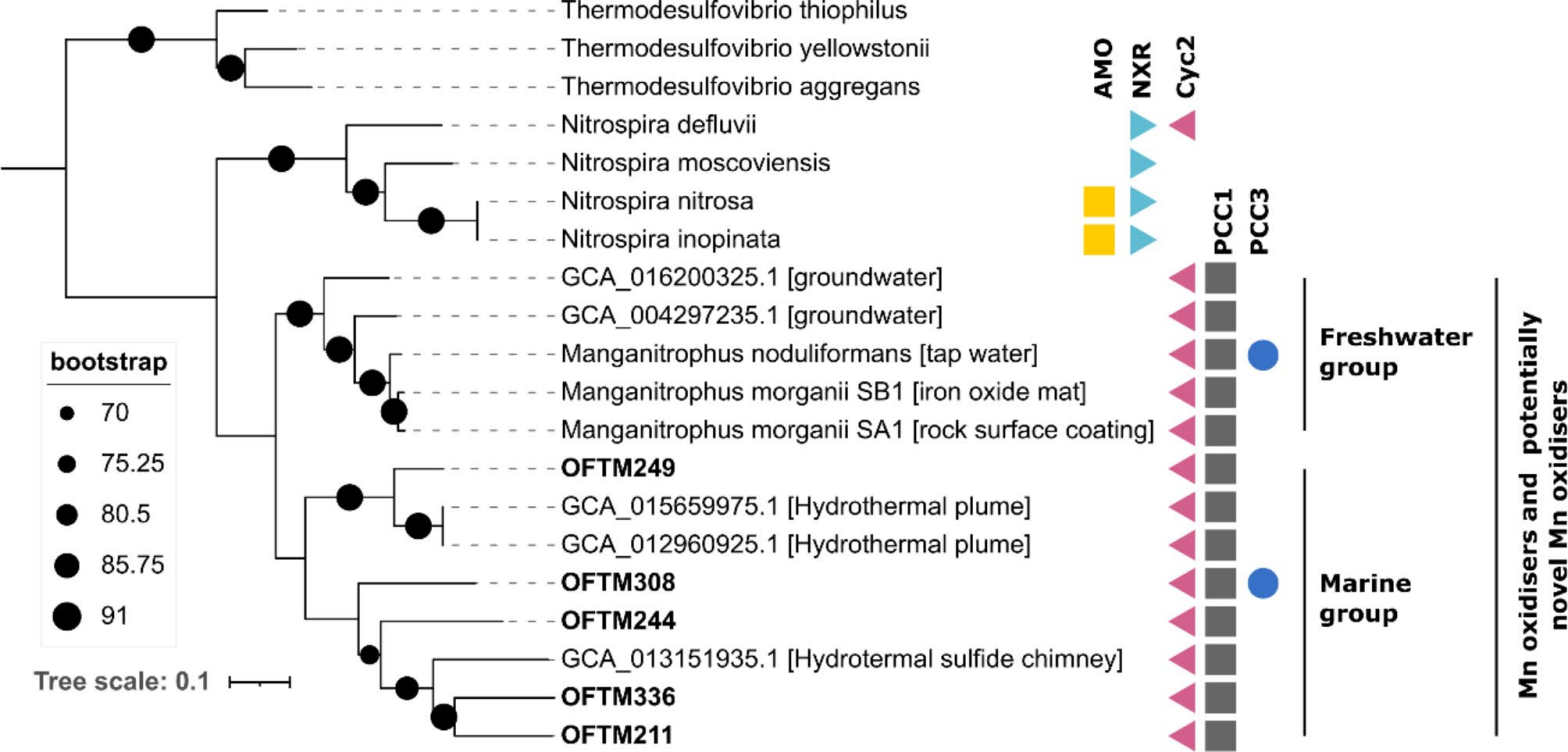
Phylogenetic tree of MAGs affiliated to the order SBBL01 based on 92 single copy genes. *Nitrospira* genomes are included as reference. *Thermodesulfovibrio* genomes were used as outgroup. Genes for enzymes involved in oxidation of nitrogen (AMO, NXR) and manganese (Cyc2, PCC1, PCCR) are indicated.

Five *Nitrospirota* MAGs affiliated to the order SBBL01 were phylogenetically related to *Candidatus* Manganitrophus noduliformans and *Ca*. Manganitrophus morganii (AAI ≥ 56%), recently discovered autotrophic manganese oxidisers^48,49^ (Fig. 7). In these bacteria, manganese oxidation is proposed to be mediated by Cyc2 and porin-cytochrome *c* (PCC) protein complexes. Cyc2 and homologues to PCC genes encoding beta barrel and multiheme *c*-type cytochrome proteins of *Ca*. Manganitrophus were identified via blastp analyses in these MAGs, while genes for nitrification-enzymes (AMO, NXR) were absent (Fig. 7). Several 16 S-based ASVs were classified as SBBL01 and varied in summed relative abundance per sample between zero and 2.4%, with an average of 0.4% and higher abundances in site P than T (DESeq2, p_adj_ < 0.01) (Figure S11). The SBBL01 MAGs from this study were phylogenetically related to genomes recovered from hydrothermal vents (AAI ≥ 60%), forming a marine clade of potentially novel manganese oxidisers, distinct from freshwater manganese oxidisers, as recently proposed^49^. However, whether homologs of manganese oxidases identified in these MAGs are implicated in the oxidation of manganese or in other reactions remains to be elucidated.

### The biofilm community contained an abundance of microorganisms with potential for nitrogen cycling

Both amplicon sequencing and metagenomics indicated high relative abundances of putative nitrifiers such as *Nitrosomonas*, *Nitrosopumilus* and *Nitrospina*, which had been previously detected in Oslofjord biofilms^21^. The most abundant populations were classified as *Nitrosopumilus* (Fig. 4C, S8), putative aerobic ammonium oxidizing Archaea based on taxonomy as well as on the identification of nitrification-related *amoABC* genes (Figure S12). Also, MAGs affiliated to marine anaerobic ammonium-oxidizing (anammox) bacteria within *Ca*. Scalindua and the newly discovered *Ca.* Anammoxibacter^22^ were occasionally detected in biofilms, at relative abundances ranging between 0 and 5.4%. In addition, genes linked to denitrification were common (Figure S12). 191 MAGs had genes to potentially reduce nitrate to nitrite (*nar/nxr* or *nap*). For subsequent denitrification steps, the abundance of denitrification genes decreased in abundance, with only 82 of the MAGs having the nitrous oxide reductase (*nosZ*). In 25 MAGs, we also observed the ammonia forming nitrite reductase gene *nrfA*, indicating a potential for dissimilatory nitrate reduction to ammonium (Figure S12).

## Discussion

Results from this study indicate that deterioration mechanisms of sprayed concrete in subsea tunnels are complex and largely differ from biodeterioration in other environments where sulfide-induced degradation and corrosion resulting from microbial sulfate reduction predominates^3^. Previous investigations have revealed that MICC in subsea road tunnels in Norway is due to a combination of biotic and abiotic processes^18,20^. Consistent with these investigations, the abiotic attack detected in our study mainly affected concrete adjacent to the rock mass, governed by infiltration of magnesium, sulfate and bicarbonate-enriched saline groundwater with a of pH ≈ 7.5-8. Based on the results shown here, it is clear that acidification and calcium leaching were caused by biomineralisation and redox reactions within the biofilm. The abiotic degradations involved thaumasite sulfate attack, magnesium attack, popcorn calcite deposition and chloride attack (Fig. 1, Figures S4-S6). Also, the near surface formation of thaumasite at a later stage at P2 and the deeper formation of M-S-H must be regarded as abiotic, yet facilitated by increased porosity due to the earlier presence of acids (Figure S4), whilst the different outer carbonates possibly represent biominerals.

Rapid biofilm formation has been previously observed after a few weeks upon concrete spraying in the Oslofjord subsea tunnel, mainly in the presence of water-bearing cracks^18^. Changes in biofilm appearance observed in this study (Figure S2) suggest shifts in microbial community composition, structure and function over time. Accordingly, our 16 S rRNA gene analyses suggest differences in community structure from 2015 to 2020. Alpha and beta diversity gradually increased from 2015 to 2020 (Fig. 3), which is in line with species-time relationships observed in many microbial ecosystems^64^, potentially reflecting interspecies interactions resulting in increased niche availability over time^65^. Similar succession dynamics were recently shown for time scales of several months on concrete exposed to seawater, where deterministic forces via niche availability were suggested to shape microbial community structure^66^. Additionally, the two tunnel localities, T and P, had dissimilar communities over the investigated period (Fig. 4, S8) despite their geographical proximity (< 200 m), suggesting contrasting local environmental conditions.

Our set of 401 MAGs allowed the reconstruction of metabolic potential in the microbial members of the Oslofjord tunnel biofilms. Based on metagenomic analyses, it was shown that the microbial communities had a strong marine signature, indicated by many marine taxa among the 186 MAGs classified to genus level, and by the observation that genes involved in osmotic stress were widespread.

Microorganisms in marine sediments persist for several kilometres below the sea floor^67,68^. Therefore, it is inferred that several Oslofjord tunnel biofilms were seeded by microorganisms from the fjord sediment above the tunnel, and possibly also from the jointed rock mass. This is supported by a recent study that revealed the presence of a novel family of anammox bacteria in Oslofjord biofilms. Meta-analysis suggests that these bacteria are present also in marine sediments^22^. The subsea biofilm community was, furthermore, a rich source of novel microorganisms, many of which could only be classified at higher taxonomic levels. This indicates that microbiomes associated with manmade structures, such as sprayed concrete, are largely unexplored having unknown genetic potential.

### Potential for sulfur oxidation predominated over potential for sulfur reduction

MICC has been extensively studied in sewer systems, where microbial degradation of organic matter leads to oxygen depletion. Sulfur reduction by sulfate-reducing bacteria (SRB) typically occurs in anaerobic environments, followed by subsequent sulfide oxidation by sulfur-oxidizing bacteria (SOB), producing sulfuric acid that deteriorates concrete^3,69–72^. In contrast to sewers, the availability of organic matter is limited in subsea tunnels, and electron acceptors such as oxygen, nitrate, iron and manganese are available to biofilms, which may hamper dissimilatory sulfate reduction^73^.

We did not detect any of the taxa typically associated to sulfur cycling in MICC, such as SOB species of *Thiobacillus* or *Acidithiobacillus*^74^. Furthermore, *Desulfobacterota* (formerly known as *Deltaproteobacteria*), a group known to encompass bacteria capable of sulfur reduction and oxidation^75^ was represented by only four MAGs with an average abundance of 0.36%. Yet, members in *Desulfobacterota* are abundant in Oslofjord sediments above the tunnel^76^, suggesting that sulfate reduction is a key pathway in this ecosystem, as typical in marine sediments^77^. This indicates that environmental conditions in the tunnel were selected against sulfate reducers. This was also confirmed by the low relative abundance (0.06%) of the only MAG having a reductive-type of the *drsAB* gene.

**Fig. 8:**
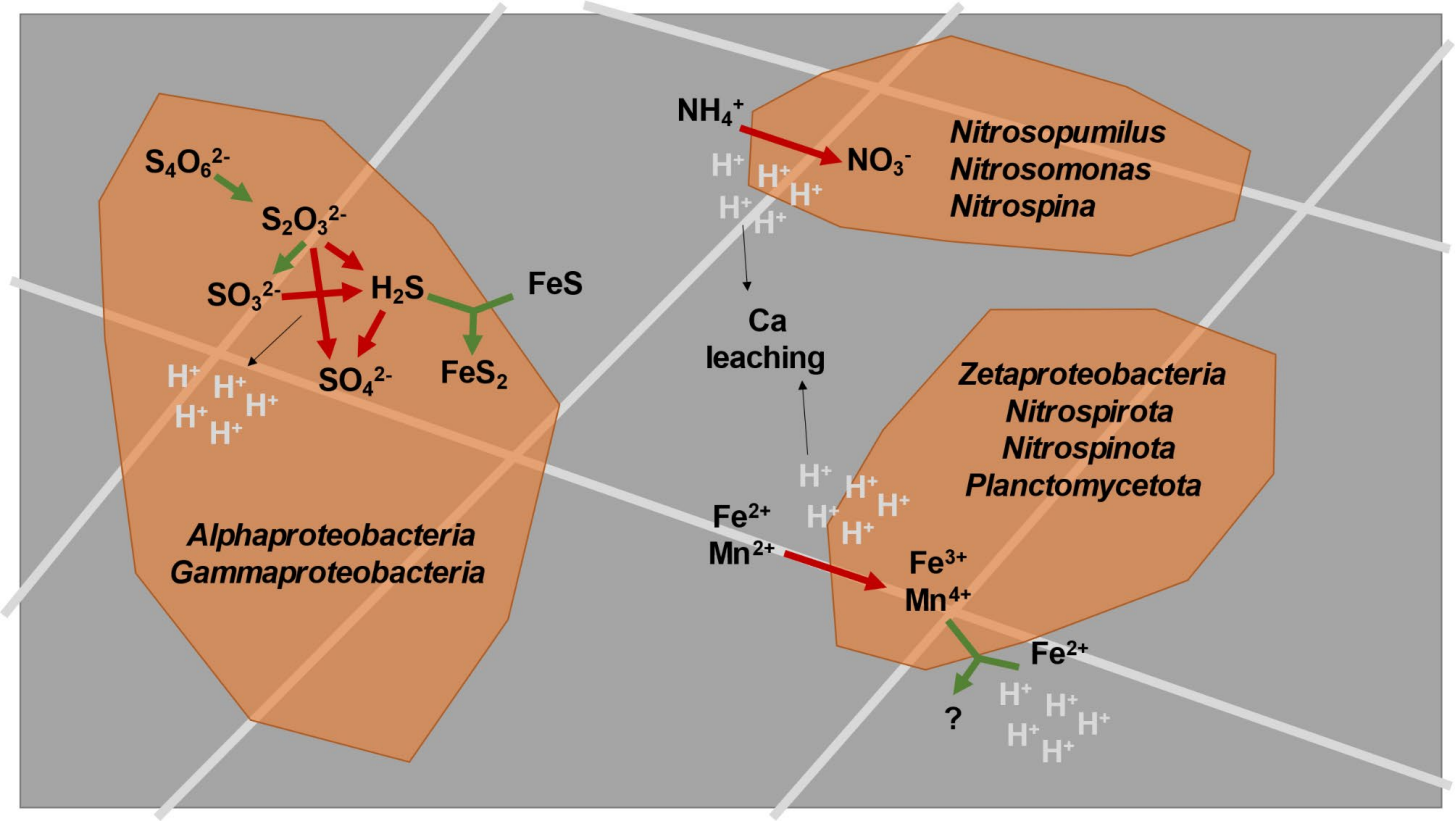
Conceptual model of the mechanisms underlying concrete biodeterioration. The colours represent: dark grey = concrete, light grey = steel fibre, orange = biofilm, green arrow = reduction, red arrow = oxidation.

Conversely, a considerable share of the biofilm community (263 out of 401 MAGs) harboured genes for sulfur oxidation (Fig. 5), including oxidative-type *dsrAB* genes as well as genes in the Sox pathway. Moreover, even the reductive-type *dsrAB*, detected may have been associated with sulfate oxidation, as recently suggested^56,78^. The potential for tetrathionate reduction to thiosulfate was detected in 52 MAGs, and thiosulfate disproportionation to sulfide and sulfite in 41 MAGs, potentially providing these substrates for sulfur oxidation in the biofilms via enzymes encoded by *soeA*, *sorA*, *dsrABC* and *sqr*, identified in this study. Sulfide-producing reactions could explain the presence of marcasite (FeS_2_) from either reduction of sulfate in biofilm water^18^ or from a combination of FeS and sulfide^79^. Furthermore, biological oxidation of FeS_2_ at circumneutral pH has been reported^80^. Given the presence of sulfur oxidation-related genes in the MAGs, sulfur oxidation could be a mechanism promoting acidification in the biofilms. Together with sulfate measured in water, this would contribute to sulfate attack in the concrete (Fig. 8).

### Iron-oxidising bacteria could deteriorate steel fibres and contributed to acidification

The orange colour (Figure S2) and previously measured high concentrations of iron in the biofilm^21^ indicate considerable formation of Fe(III) oxides (i.e. ferrihydrite). The steel fibres apparently supported extensive iron oxidation. Under circumneutral pH and atmospheric oxygen concentrations, abiotic oxidation of ferrous iron occurs rapidly, forming Fe(III) oxides that are poorly soluble and outcompete biotic Fe(II) oxidation. However, at microaerophilic conditions, typical in inner layers of biofilms, microbial oxidation can compete with abiotic Fe(II) oxidation^59,81^.

SEM observations of bacteria in the biofilms associated with Fe (Fig. 1) indicate a biological role for the observed iron oxidation. *Zetaproteobacteria* had been previously observed at the Oslofjord biofilms and were presumed to be the main iron oxidizers in the biofilms^21^. *Zetaproteobacteria* are considered model organisms for marine microbial Fe(II) oxidation, typically found in sediments^59,82^. They have also been found in saline terrestrial springs and biofilms associated with steel corrosion^83^. However, among the MAGs, a wide distribution of genes linked to iron oxidation was identified. Apart from *Zetaproteobacteria*, such genes were detected in other *Proteobacteria*, *Nitrospirota*, *Nitrospinota* and *Planctomycetota*, indicating widespread potential for iron oxidation. Higher relative abundances of *Zetaproteobacteria* at site P (Fig. 6C) corresponded to a more reddish colour of the P biofilm (Figure S2). A higher water flow rate at site P may result in a higher oxygen penetration into the biofilm due to a smaller boundary layer^21^, which could favour aerobic processes. The presence of iron-oxidising bacteria together with the observed corrosion of steel fibres further support the observed biodeterioration of concrete due to acidification caused by biotic Fe(II) oxidation.

### **Biological manganese oxidation likely contributed to MICC**

In the tunnel biofilm, nodules of Mn oxides were detected and on the outer areas of the concrete, Mn oxides were present as buserite and todorokite (Table S4-S6, Fig. 1). Previous measurements have also shown that the inflowing water provided a steady source of manganese^21^. Mn-oxides are, next to oxygen, one of the strongest oxidisers in the environment^52^. Heterotrophic bacteria capable of Mn oxidation, as revealed by cultivation of isolates, are found among many phyla^52,84^ and in metagenomes, several genes have been detected with putative roles in Mn oxidation^85,86^. The mechanisms for Mn oxidation by heterotrophic bacteria, as well as the physiological reasons for doing it, are however complex and yet not resolved^84^. Previous characterisation of the elemental composition of the biofilm also showed high Mn concentrations, although no canonical heterotrophic Mn oxidizing bacterium was observed by amplicon sequencing of the 16 S rRNA gene^21^.

The first autotrophic Mn-oxidising bacterium, *Manganitrophus noduliformans* has been recently described. This bacterium, within the phylum *Nitrospirota*, originating from freshwater, gains energy from Mn(II) oxidation using oxygen for respiration and forms nodules of Mn(IV) oxide^48^. In the subsea tunnel biofilms, several MAGs affiliated to a *Manganitrophus*-like cluster were identified (Fig. 7). The environmental distribution of bacteria related *Manganitrophus* is broad^48,49^ and this study demonstrates that they are indeed present in saline environments. These MAGs likely represent bacteria that oxidise Mn since they contain the genes proposed to be involved in Mn oxidation. As *Manganitrophus*-like MAGs were present in every biofilm sample (average relative abundance of 1%), the activity of these populations may explain the formation of Mn oxide nodules and Mn biominerals such as buserite and todorokite detected in the Oslofjord subsea tunnel. Further abiotic reduction of Mn(IV) oxide with concomitant oxidation of ferrous iron (Fe^2+^), which is an important reaction in marine and freshwater sediments^87^, may have participated in acidification in the deeper parts of the biofilm, as previously suggested^18^.

### Nitrification was likely an important mechanism for concrete deterioration

It was estimated that putative nitrifiers such as *Nitrosopumilus*, *Nitrosomonas*, and *Nitrospina* were the most abundant community members of biofilms based on 16 S- and genome-calculated relative abundances (Fig. 4 and S8). Therefore, we infer that ammonium oxidation was a key process in this ecosystem, likely causing concrete deterioration via the release of protons, leading to acidification of the biofilm porewater. This could have contributed to the measured leaching of Ca from the concrete and the associated impairment of its structural properties, along with the effects from Fe and Mn oxidation (Fig. 1, Table S1-S3, Fig S2-S5).

Ammonium concentrations in water flowing over the biofilms have been shown to be higher (< 0.3–1.2 mg N l^− 1^, Table S2-3) than in the water column in the Oslofjord at 60 m depth (< 0.005 mg l^− 1^)^18^. This suggests that, in the sediments above the tunnel, ammonium was released from organic matter degradation, as typical for marine sediments including the Oslofjord^76^. Catalytic converters in motor vehicles that traffic through the tunnel could be a potential source of ammonium^88^. However, this is likely a minor source in this case because all sample sites were isolated from the road traffic by thick concrete shields.

Furthermore, MAGs capable of heterotrophic denitrification and anammox were observed in microbiomes^22^ (Figure S12). This indicates a potential for the turnover of nitrogen from water to air in the microbiomes, and the use of nitrogen as an alternate electron acceptor, with denitrification potentially having a role in corrosion^89^.

## Conclusions

Here we identified potential mechanisms for concrete biodeterioration that largely differ from degradation processes taking place in other environments. We propose that the key acidifying activities causing concrete biodeterioration included the biogenic oxidation of ammonium, several sulfur compounds, iron and manganese minerals (Fig. 8). Such oxidative reactions may furthermore have resulted in steel corrosion and sulfate production within the biofilm, contributing to sulfate attack on the concrete. To get a full understanding of the relative importance of the various microbial transformations for the concrete degradation, future manipulative studies are, however, necessary.

Our data suggests that acidification within the biofilms resulting in biodeterioration was restricted to situations with low water flow. The sprayed concrete at site P2 showed deep effects of biodeterioration after less than five years of low water flow, with later aggravation of the concrete at higher flow. In contrast, site P1 with higher flow showed only marginal deterioration during seven years, even though the microbial communities were similar between the sites. This is likely because the saline groundwater neutralized the acid producing reactions within the biofilm. Also, site T with a history of low water flow showed clear effects of biodeterioration.

Long-term considerations in connection with the maintenance of sprayed concrete rock reinforcement in subsea tunnels should include monitoring of the pH, fluctuating water flow, sizes of biofilms and with focus on locating loose deteriorated concrete.

## Electronic supplementary material

Below is the link to the electronic supplementary material.

Supplementary Material 1

## Data Availability

Raw metagenome reads and MAGs have been deposited to the NCBI under the BioProject PRJNA755678 and raw sequences for the 16 S rRNA genes under the Bioproject PRJNA481470, accession numbers SAMN09669680-SAMN09669744 and PRJNA1061464, accession numbers SAMN39270767-SAMN39270823. Files with taxonomy and relative abundance of MAGs, FeGenie results, DiSCo results and DRAM genomes annotations have been deposited in Zenodo (DOI: 10.5281/zenodo.10406297).
