# Supplementary material for: Microbial acidification by N, S, Fe and Mn oxidation as a key mechanism for deterioration of subsea tunnel sprayed concrete
Source: Sci Rep. 2024 Sep 30;14:22742. doi: 10.1038/s41598-024-73911-w (PMC11442690; doi:10.1038/s41598-024-73911-w)
Supplement: Supplementary file 1 — Supplementary Material 1 [file 41598_2024_73911_MOESM1_ESM.docx]

Supplementary information

**Microbial acidification by N, S, Fe and Mn oxidation as a key mechanism for deterioration of subsea tunnel sprayed concrete**

Sabina Karačić ^1,5#^, Carolina Suarez ^2,7#^, Per Hagelia ^3,6^, Frank Persson ^1^, Oskar Modin ^1^, Paula Dalcin Martins ^4,8^, Britt-Marie Wilén ^1^*

^1^ Department of Architecture and Civil Engineering, Chalmers University of Technology, Göteborg, Sweden.

^2^ Division of Water Resources Engineering, Faculty of Engineering LTH, Lund University, Lund, Sweden.

^3^ Construction Division, The Norwegian Public Roads Administration, Oslo, Norway.

^4^ Department of Ecosystem and Landscape Dynamics, University of Amsterdam, Amsterdam, Netherlands.

^5^ Institute of Medical Microbiology, Immunology and Parasitology, Medical Faculty, Rheinische Friedrich-Wilhelms Universität, 53127, Bonn, Germany

^6^Müller-Sars Biological Station, Ørje, NO-1871, Norway

^7^Sweden Water Research AB, Lund, 222 35, Sweden

^8^Microbial Ecology Cluster, GELIFES, University of Groningen, Groningen, 9747 AG, Netherlands

# Tables

**Table S1.** Summary of chemical composition of water that flowed over areas with concrete and biofilm (WCB) and water that flowed through rock mass and concrete cracks without contact with outer biofilm (WR) at the Pump station location P1.

| **P1** | **Year** | **2016** | | **2019** | |
| --- | --- | --- | --- | --- | --- |
|  | **Water type** | **WCB** | **WR** | **WCB** | **WR** |
| Mg  Al  Ca  Ti  V  Cr  Mn  Fe  Co  Ni  Cu  Zn  Cd  Pb | mg l^-1^  mg l^-1^  mg l^-1^  mg l^-1^  mg l^-1^  mg l^-1^  mg l^-1^  mg l^-1^  mg l^-1^  mg l^-1^  mg l^-1^  mg l^-1^  mg l^-1^  mg l^-1^ | 1,032  0.620  389  0.899  0.26  0.010  1.52  0.01  0.002  0.821  0.382  1.091  0.001  0.042 | 1,147  0.673  292  0.690  0.121  0.007  1.17  0.01  0.002  0.221  0.184  2.899  0.002  0.163 | 1,108  0.401  557  0.729  0.142  0.008  1.47  0.01  0.002  0.775  0.296  1.118  0.001  0.050 | 1,202  0.378  264  0.660  0.124  0.007  1.39  0.01  0.003  0.823  0.401  4.929  0.002  0.216 |

**Table S2.** Summary of chemical composition of water that flowed over areas with concrete and biofilm (WCB) and water that flowed through rock mass and concrete cracks without contact with outer biofilm (WR) at the Pump station location P2.

| **P2** | **Year** | **2015** | | **2016** | | **2017** | | **2019** | | **2020** | |
| --- | --- | --- | --- | --- | --- | --- | --- | --- | --- | --- | --- |
|  | **Water type** | **WCB** | **WR** | **WCB** | **WR** | **WCB** | **WR** | **WCB** | **WR** | **WCB** | **WR** |
| Mg  Al  Ca  Ti  V  Cr  Mn  Fe  Co  Ni  Cu  Zn  Cd  Pb  Cl^-^  SO_4_^2-^  NH_4_^-^  NO_3_^-^  Alkalinity  pH  DOC | mg l^-1^  mg l^-1^  mg l^-1^  mg l^-1^  mg l^-1^  mg l^-1^  mg l^-1^  mg l^-1^  mg l^-1^  mg l^-1^  mg l^-1^  mg l^-1^  mg l^-1^  mg l^-1^  mg l^-1^  mg l^-1^  mg l^-^  mg l l^-1^  -  mg l^-1^ | 2,232  0.356  604  0.96  0.26  0.015  1.32  0.01  0.003  0.747  0.42  1.025  0.001  0.032 | 1,147  0.673  292  0.848  0.127  0.009  1.28  0.01  0.002  0.247  0.266  0.801  0.002  0.037 | 1,323  0.319  343  1.009  0.146  0.011  1.31  0.01  0.002  0.784  0.337  1.072  0.001  0.039  22,400  3,260  <0.32  3.02  2.28  7.55  <1.50 | 1,202  0.378  297  0.887  0.132  0.212  1.28  0.01  0.004  0.942  0.398  5.983  0.002  0.082  20,000  2,700  0.74  <1.25  2.3  7.5 | 1,150  0.328  274  0.815  0.126  0.007  1.03  0.01  0.001  0.809  0.294  1.141  0.001  0.048 | 821  0.352  236  0.783  0.114  0.007  1.28  0.01  0.005  1.198  0.462  3.382  0.002  0.172 | 1,110  0.412  560  0.737  0.121  0.008  1.46  0.01  0.002  0.811  0.347  1.034  0.001  0.049  20,000  2,660  0.772  <1.25  3.12  7.7  1.3 | 1,120  0.366  453  0.711  0.107  0.007  1.47  0.01  0.003  1.692  0.484  4.329  0.002  0.176  19,300  2,550  1.21  <1.25  2.16  7.7  3.6 | 1,253  0.457  563  0.702  0.174  0.007  1.35  0.1  0.002  0.832  0.296  1.282  0.001  0.055 | 1,281  0.391  287  0.683  0.119  0.006  1.41  0.01  0.004  1.712  0.492  5.337  0.002  0.989 |

**Table S3.** Summary of chemical composition of water that flowed over areas with concrete and biofilm (WCB) and water that flowed through rock mass and concrete cracks without contact with outer biofilm (WR) at the Test site location.

| **T** | **Year** | **2016** | | **2017** | | **2019** | | **2020** | |
| --- | --- | --- | --- | --- | --- | --- | --- | --- | --- |
|  | **Water type** | **WCB** | **WR** | **WCB** | **WR** | **WCB** | **WR** | **WCB** | **WR** |
| Mg  Al  Ca  Ti  V  Cr  Mn  Fe  Co  Ni  Cu  Zn  Cd  Pb  Cl^-^  SO_4_^2-^  NH_4_^-^  NO_3_^-^  Alkalinity  pH  DOC | mg l^-1^  mg l^-1^  mg l^-1^  mg l^-1^  mg l^-1^  mg l^-1^  mg l^-1^  mg l^-1^  mg l^-1^  mg l^-1^  mg l^-1^  mg l^-1^  mg l^-1^  mg l^-1^  mg l^-1^  mg l^-1^  mg l^-^  mg l l^-1^  -  mg l^-1^ | 1,416  0.646  362  0.993  0.16  0.016  1.36  0.02  0.003  1.559  0.495  3.377  0.002  0.097  19,000  2,600  1.1  <1.25  2.4  7.6 | 1,400  0.381  314  0.898  0.155  0.01  1.54  0.02  0.002  1.229  0.476  3.249  0.002  0.125 | 1,121  0.531  384  0.618  0.144  0.01  2.11  0.17  0.002  0.004  0.233  0.091  0.006  0.068 | 1,224  0.392  361  0.675  0.158  0.01  1.7  0.17  0.003  1.161  0.456  3.241  0.004  0.167 | 1,358  0.562  432  0.602  0.168  0.01  2.27  0.01  0.003  0.784  0.328  1.236  0.005  0.087 | 1,445  0.424  391  0.732  0.139  0.01  1.71  0.01  0.002  1.652  0.391  3.237  0.004  0.171 | 1,327  0.627  467  0.713  0.147  0.01  2.46  0.01  0.003  0.923  0.346  1.104  0.006  0.088 | 1,421  0.409  398  0.819  0.108  0.01  2.13  0.01  0.003  1.792  0.451  4.216  0.004  0.216 |

**Table S4:** Results from small sample chips of outermost concrete with biominerals analysed with SEM imaging and EDS in 2020. These samples overlap with outer parts of the thin sections (Table S4). The locations have different historical developments: the concretes at P1/P2 and T were 21 years and 10 years old, respectively. Biofilms developed soon after concrete emplacement, except for at subsite P1 where biofilm growth started in newly established leakages after the water flow had increased in 2013.

|  | **P1** | **P2** | **T** |
| --- | --- | --- | --- |
| **Thickness** | 4 mm | 3 mm | 5 mm |
| **SEM** | Outer Fe-oxide nodules and Fe-rich fibrous to twisted stalks on Mn-oxide nodules and gypsum outside leached cement paste | Fe-oxide and Mn-oxide nodules on severely leached cement paste | Fe-oxide and Mn-oxides  on fibrous Ca-carbonate  with Mg-carbonate on leached cement paste |
| **Damage** | Friable concrete  Steel fibre corrosion restricted to biofilm and leached concrete with carbonate | Very friable with loss of outer concrete  Steel fibre corrosion restricted to biofilm and leached concrete with carbonate | Friable concrete  Steel fibre corrosion restricted to biofilm and leached concrete with carbonate |

**Table S5:** Results from thin sections of concrete cores analysed with polarized light and SEM-EDS in 2020 and thickness of zones. Layering of outer rather soft Fe-rich biofilm on Mn-oxides was lost in thin section preparation. The concretes at P1/P2 and T were 21 years and 10 years old, respectively. Biofilms developed soon after concrete emplacement, except for at subsite P1 where biofilm growth started in newly established leakages after the water flow had increased in 2013.

|  | **P1** | **P2** | **T** |
| --- | --- | --- | --- |
| **Outer deposits** | Mn-micro nodules outside Ca-Mg-carbonate (0-100 µm) | Mn-oxide deposits outside carbonate deposits and Mg substituted paste (M-S-H) (100-1000 µm) | Mn-oxide outside carbonate and brucite (0-100 µm) |
| **Concrete zonation** | 1) Carbonated porous paste with air voids (5 mm):  - air voids in outer parts with deposits of Ca, Si, Mg-carbonate ± Mn-oxide  - air voids further inside with only brucite  2) Inner more sound concrete with empty air voids (25 mm):  - some Mg & S substitution with shrinkage cracks  - steel fibre mainly intact; occasionally marginally corroded | 1) Acid-leached very porous zone with Ca-depleted paste (substituted by Mg, S) and extensive dissolution of pre-existing thaumasite (10-15 mm)  -steel fibre corrosion  2) Inner concrete with Ca-depleted paste, M-S-H and significant shrinkage cracks (25 mm):  - steel fibres mainly intact | 1) Outer Ca-depleted paste (2-5 mm):  - mainly M-S-H, with PCD (calcite) and brucite  - Steel fibre corroded only next to PCD  2) Internal zone completely transformed into M-S-H and loss of Ca (30 mm):  - PP-fibre sometimes extensively split by ettringite growth  3) Inner concrete with empty air voids near rock adhesion zone (4-8 mm):  - Ca-depleted paste with a little Mg, S, PCD and thaumasite attack |

**Table S6:** Results from SEM and XRD analyses of outer biofilm in 2020. The locations have different historical developments: The concretes at P1/P2 and T were 21 years and 10 years old, respectively. Biofilms developed soon after concrete emplacement, except for at subsite P1 where biofilm growth started in newly established leakages after the water flow had increased in 2013.

|  | **P1** | **P2** | **T** |
| --- | --- | --- | --- |
| **XRD** | Halite, buserite, todorokite  amorphous ferrihydrite | Halite, buserite, todorokite, gypsum, marcasite, dickite, amorphous ferrihydrite | Quartz, buserite, todorokite, muscovite and amorphous ferrihydrite |
| **SEM** | Soft Mn-globules with minor presence of Fe-rich fibrous filaments on halite | Fe-rich fibrous filaments and twisted stalks and Mn-globules | Fe-rich fibrous filaments and twisted stalks |

**Table S7:** Location characteristics in 2020. The locations have different historical developments: P1 was 7 years old, P2 was ca 21 years old and T was 10 years old.

|  | **P1** | **P2** | **T** |
| --- | --- | --- | --- |
| **Biofilm colour** | yellowish-brown & black  (layered) | rusty brown & black (layered) | rusty brown and black (layered) |
| **Biofilm age** | 2013 to 2020 | 1999 to 2020 | 2010 to 2020 |
| **pH/Eh (ORP)** | 6.65 / + 70 mV | 6.85 / + 158 mV | 7.20/ + 85 mV |
| **Temperature** | 13 C^o^ | 13 C^o^ | 9 C^o^ |

**Table S8:** Number of biofilm samples for microbial analyses at sites P1, P2 and T in the study period. The concretes at P1/P2 and T were 21 years and 10 years old, respectively. Biofilms developed soon after concrete emplacement, except for at subsite P1 where biofilm growth started in newly established leakages after the water flow had increased in 2013.

|  | 2015 | 2016 2016 | | 2017 | 2019 | 2020 |
| --- | --- | --- | --- | --- | --- | --- |
|  | November | March | October | April | June | September |
| P1 | 2 | 5 | 6 | 4 | 4 | 4 |
| P2 | 4 | 7 | 8 | 11 | 8 | 17 |
| T | 10 | 6 | 7 | 8 | 10 | 8 |

# Figures

|  |  |  |
| --- | --- | --- |
|  |  |  |
|  |  |  |
|  |  |  |


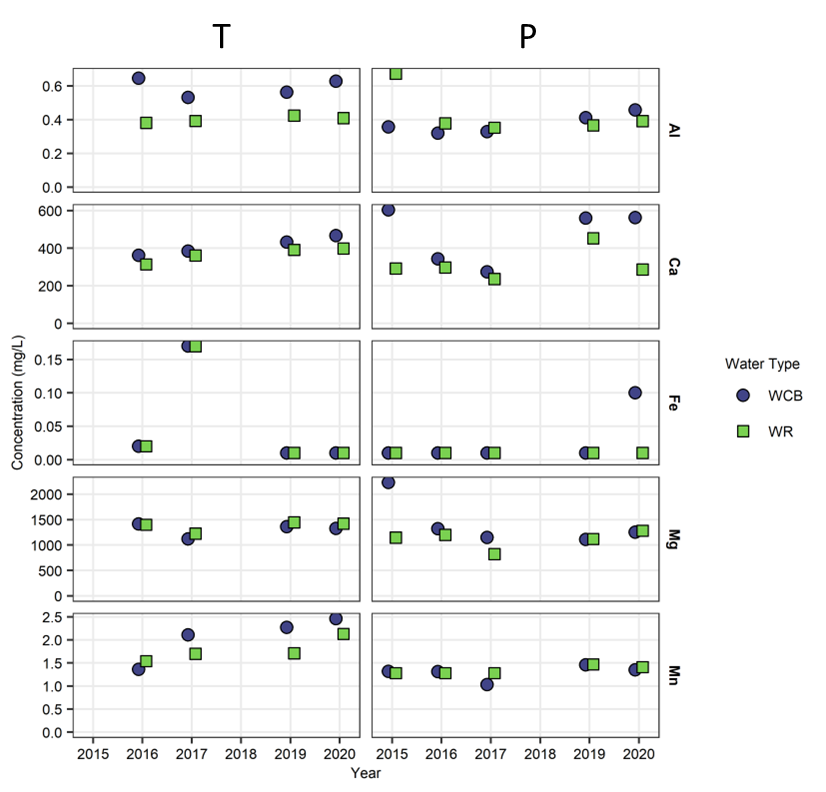


**Figure S1**. Temporal variation in water compositions at each site; water in contact with concrete and biofilm (WCB), and water without contact with biofilm (WR).

**
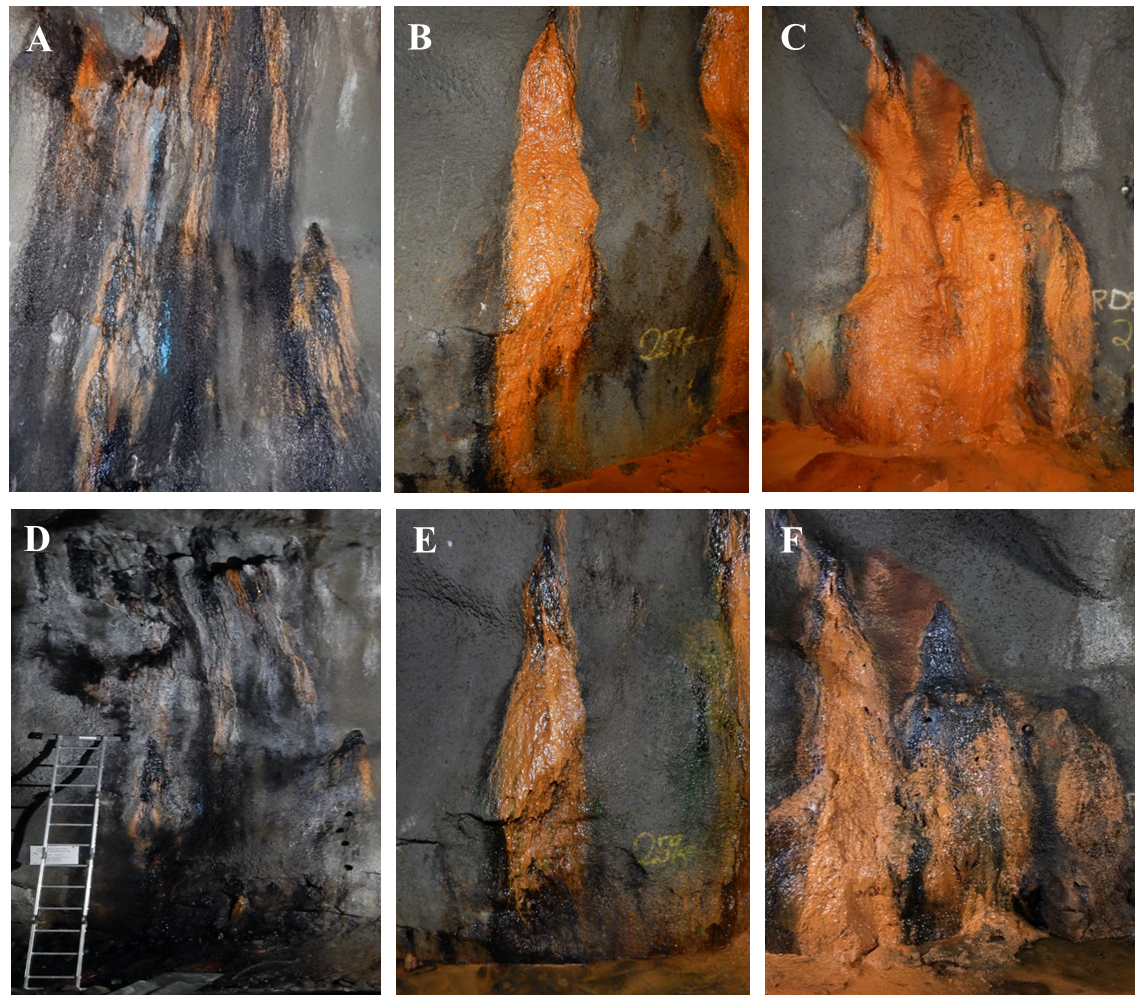
**

**Figure S2**. The biofilm appearance at sites of sampling in September 2015 (A-C) and September 2020 (D-F). Test site (A, D) and Pump station at substation P1 (B, E) and Pumpstation at substation P2 (C, F). The water flow in 2015 had been relatively high since 2013 (B) and onward, but had dropped between 2019 and 2020.


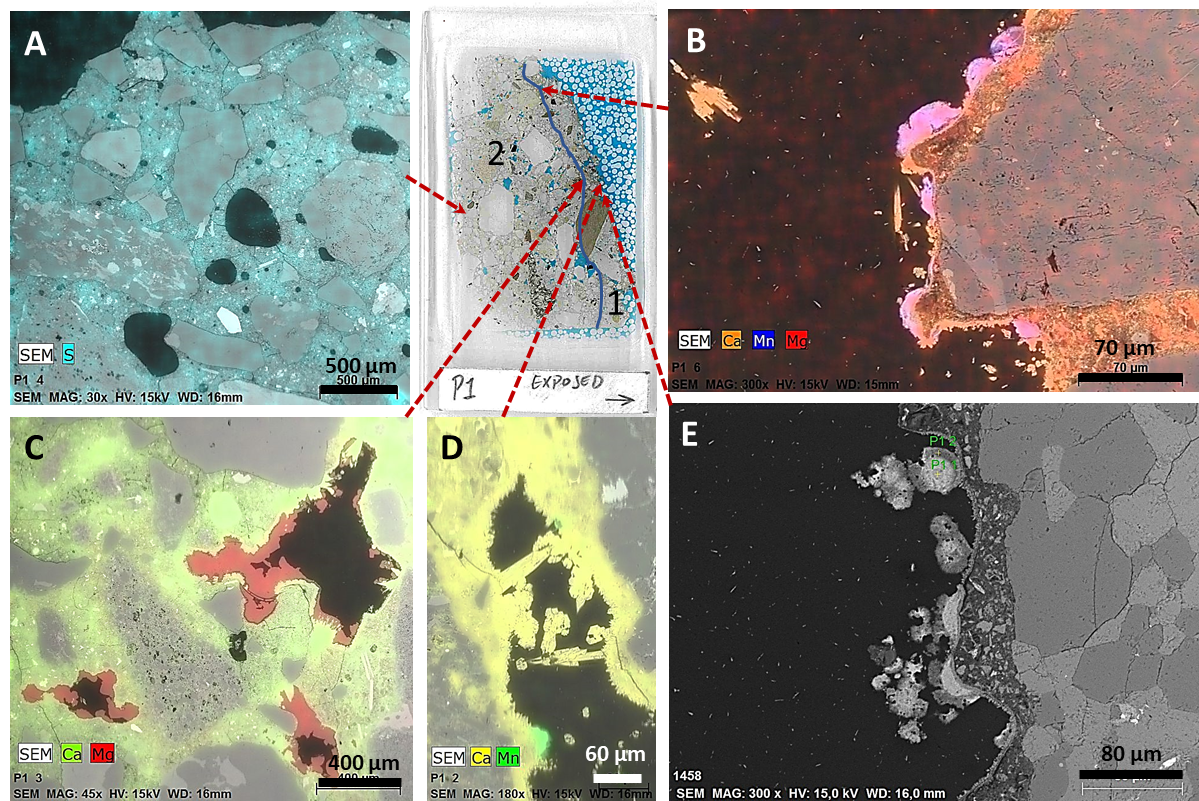


**Figure S3**. Thin section P1 with characteristic zonation and porosity shown with blue dye). Concrete age 21 years and biofilm age about 7 years. *Zone 1*: Outer parts characterised by Mn-oxide (with Mg) deposited outside carbonated cement paste (B, E) and carbonate deposits on surface-near air void with Mn-oxide. (D). Outermost Fe-rich slimy biofilm was not preserved in the thin section. *Zone 2:* Inner domain with calcium-depleted paste partly substituted by magnesium (not shown) and sulfur (A). Brucite was deposited in air voids at the transition zone between 1 and 2 (C). The overall features suggest an inward increasing pH from the surface.


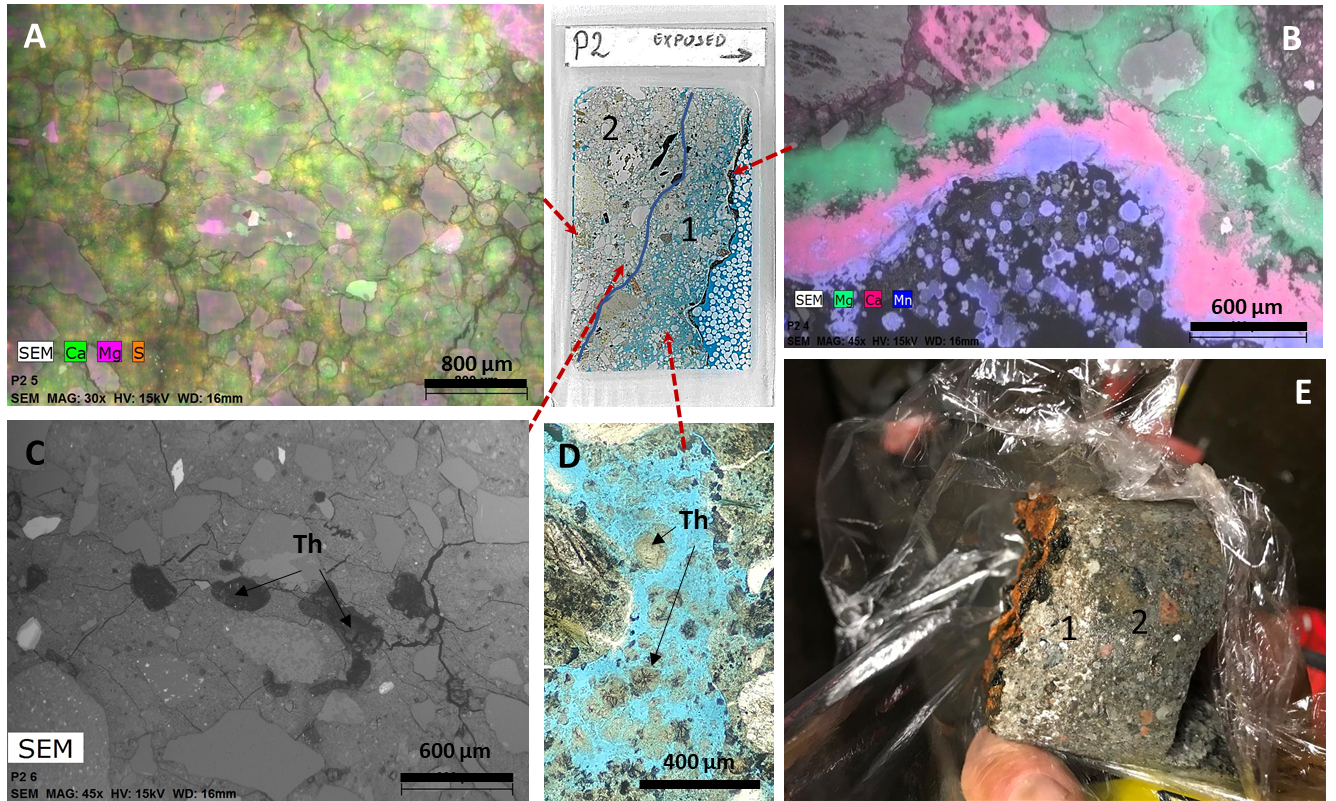


**Figure S4**. Thin section P2 with characteristic zonation and porosity shown with blue dye. Concrete and biofilm age about 21 years. *Zone 1:* Deposits of outermost Mn-oxide underneath Fe-rich slimy biofilm (not preserved in the thin section) with deposits of calcium carbonate derived from leaching of the cement paste, and inner magnesium silicate hydrate (M-S-H) (B). Most of Zone 1 was extremely porous, characterised by extensive dissolution of pre-existing thaumasite (Th) (D: plane polarised light with blue dye showing high secondary porosity). *Zone 2:* Ca-depleted cement paste substituted by Mg and S with extensive secondary shrinkage cracks (A) and occurrence of intact thaumasite (Th) in air voids towards Zone 1 (C). Image E shows the sprayed concrete core with layered Fe and Mn biominerals outside friable and leached outer Zone 1 and inner sonder Zone 2. The overall features suggest an inward increasing pH from the surface and loss of outer material due to effects from biofilm.


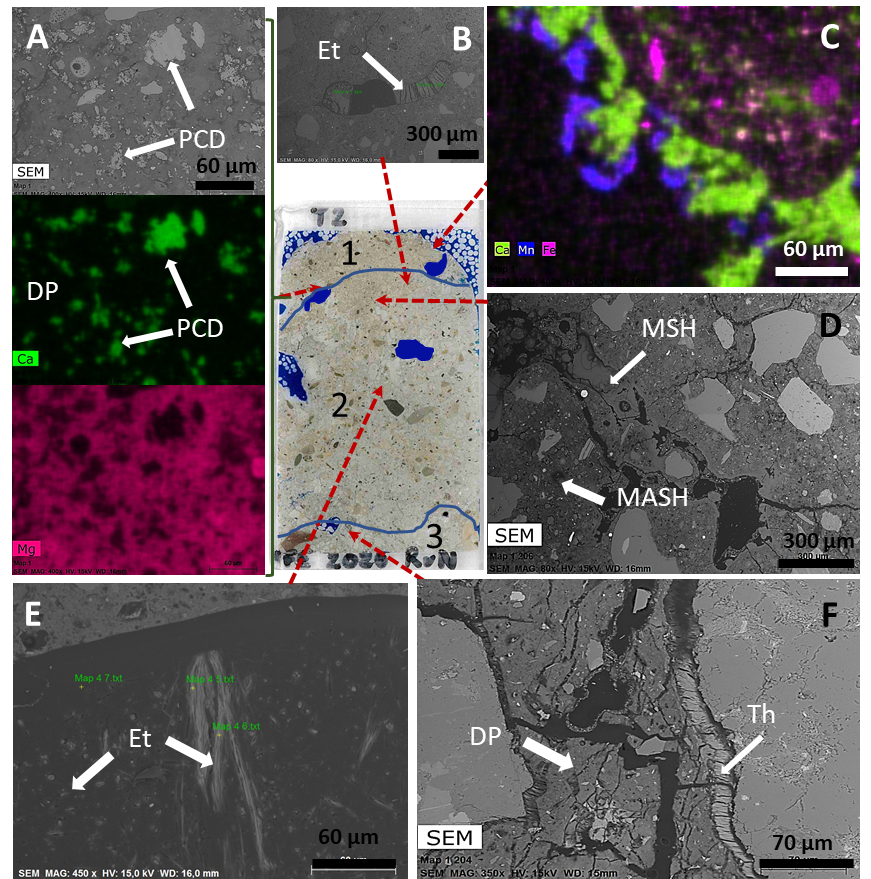


**Figure S5**. Thin section T with characteristic zonation and porosity shown with blue dye. Concrete and biofilm age 10 years. *Zone 1:* Outer leached zone with extensive Ca-depletion (DP), magnesium substitution of cement paste and Popcorn calcite deposition (PCD) (A). Ettringite deposition in air voids (B). Outermost scattered Mn-oxide underneath Fe-biofilm (not preserved in thin section) with carbonate deposits formed after leaching of the cement paste (C). *Zone 2:* Middle zone with strongly Ca-depleted cement paste being transformed into magnesium-silicate-hydrate (M-S-H) and aluminium bearing phase (“MASH”) within M-S-H (D). Growth of ettringite within polypropylene fibre, involving mechanical weakening by splitting of the fibre (E). *Zone 3:* Inner degraded zone near the contact zone with rock mass with Ca-depleted paste with a little Mg and S (DP,) and thaumasite sulfate attack (Th) (F). The overall features suggest an inward increasing pH from the surface.


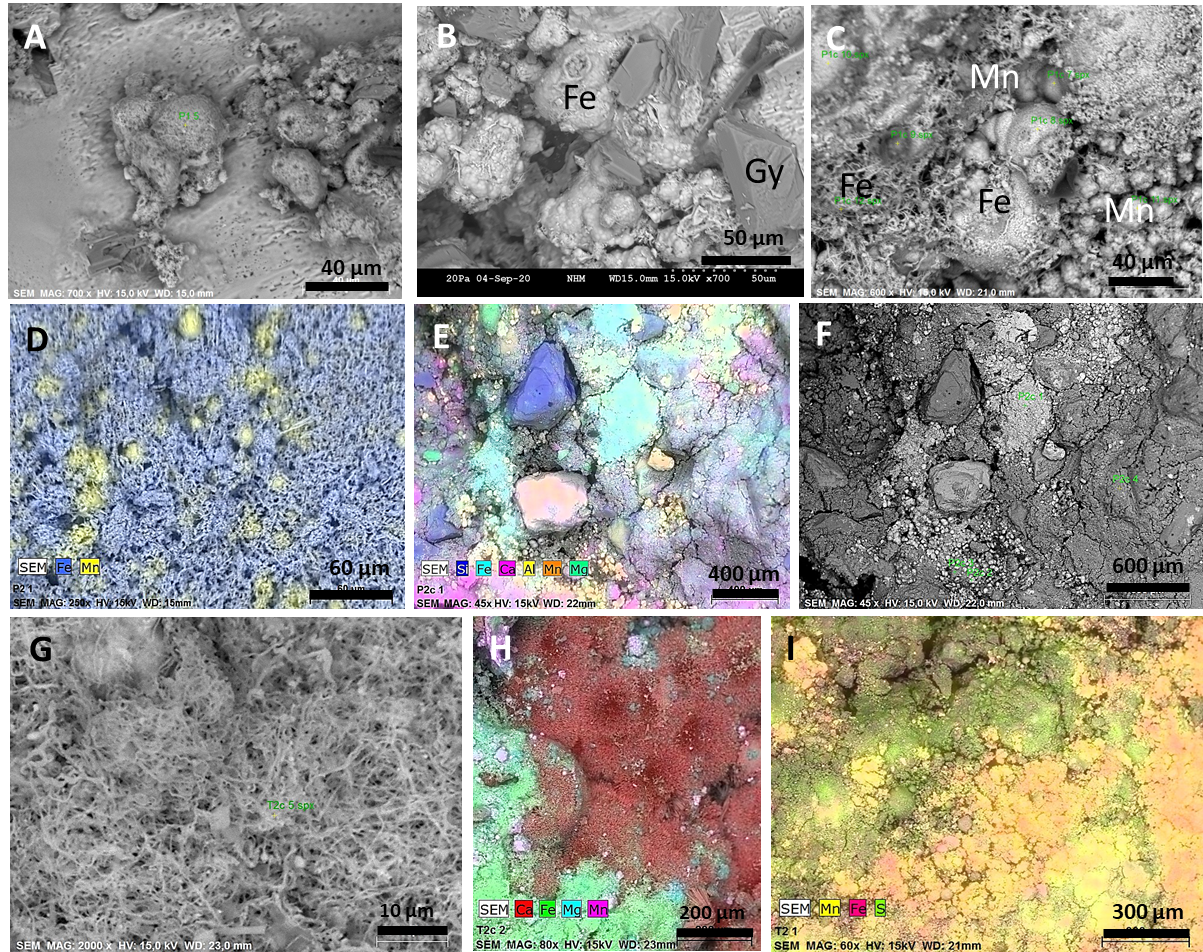


**Figure S6**. Scanning electron images and X-ray element maps of biofilm (left images) and deposits on outer concrete chips with leached cement pastes. *Sample P1:* Mn-biofilm (globules) sitting on a substrate of pitted NaCl with minor presence of Fe-rich fibrous filaments (A). Deposits of Fe-rich nodules (Fe), and gypsum (Gy) on degraded sprayed concrete (B). Fe-rich fibrous filaments with twisted stalks outside Fe-rich nodules and Mn-nodules (C). *Sample P2:* Bacterial slime with Mn-rich bacterial globules sitting within Fe-rich bacterial stalks (partly twisted) (D). Outer surface of severely leached sprayed concrete with exposed aggregate particles and scattered Fe-rich biominerals and Mn-rich micronodules (E and F). *Sample T:* Fibrous Fe-rich twisted stalks (with Si, Ca, Mn) and other microbes in outermost slimy biofilm (G). Acicular Ca-rich carbonate and Fe-rich phase (ferrihydrite-like) with scattered Mn-oxide and minor Mg-rich carbonate (H). Fe and Mn-oxide with S-rich domains at the interface between biofilm and sprayed concrete (I). Additional presence of Cl and C were detected in all samples.


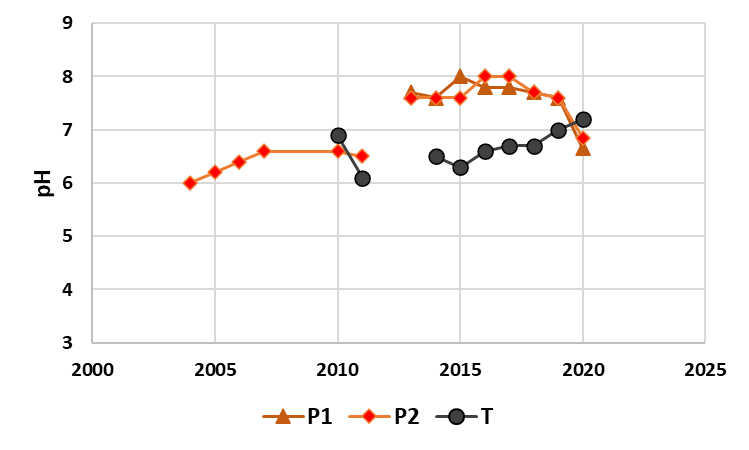


**Figure S7**. *In situ* measurements of pH in biofilm waters at the three investigated sites over time. The mildly acidic pH was associated with low water flow, whilst the higher pH similar to ground water was mainly found in events of high water flow. Each point is the average of 2-3 measurements: sometimes the pH dropped downstream within biofilm, from circumneutral to 5.8-6.2. The biofilm pH reflected a dynamic system even during short intervals, with episodes with pH = 5.5 in 2004 and early 2005. Data from (Hagelia, 2011)and unpublished from the Norwegian Public Roads Administration.

Reference: Hagelia, P., Deterioration mechanisms and durability of sprayed concrete for rock support in tunnels. PhD thesis. TU Delft, Delft, the Netherlands (2011).


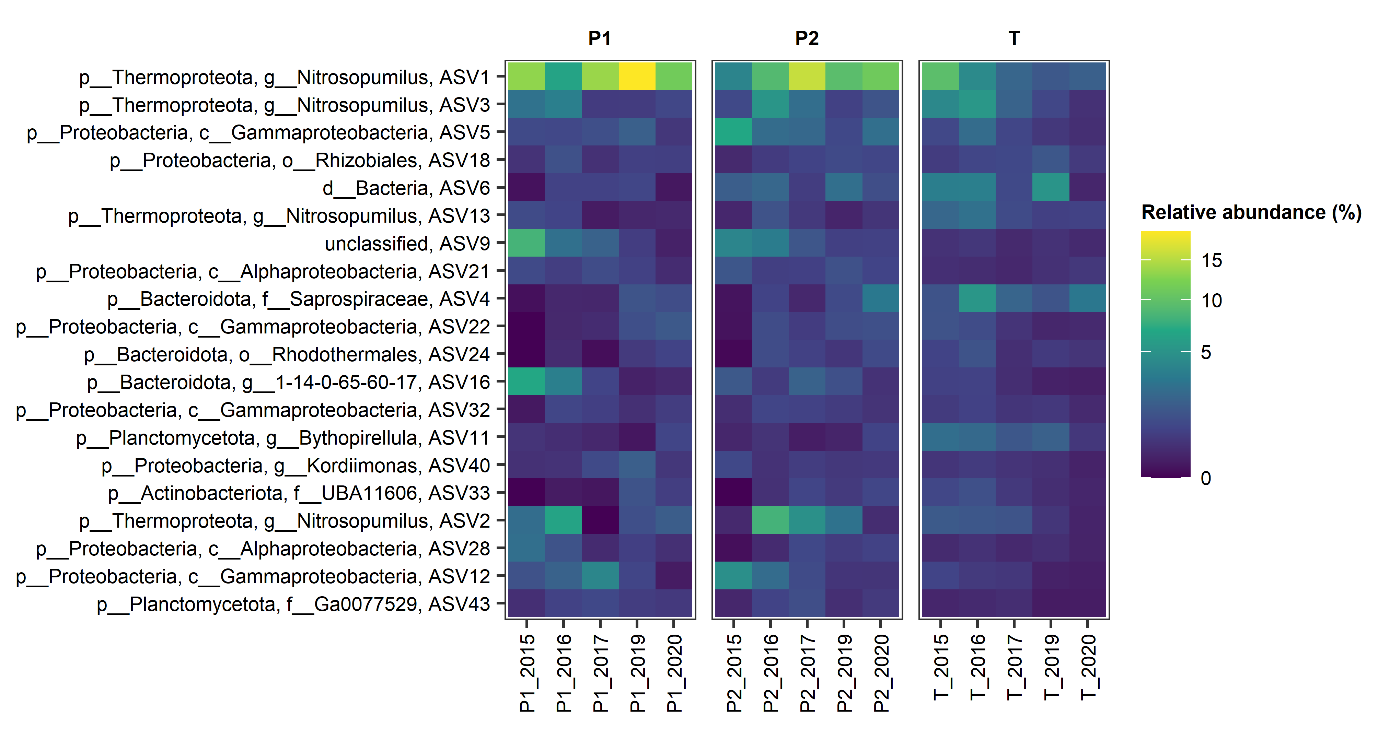


**Figure S8**. Heatmap of the top 20 ASVs in the biofilm over the years 2015-2020. For each location and year, the average of the ASV across different spots is shown in the heatmap.


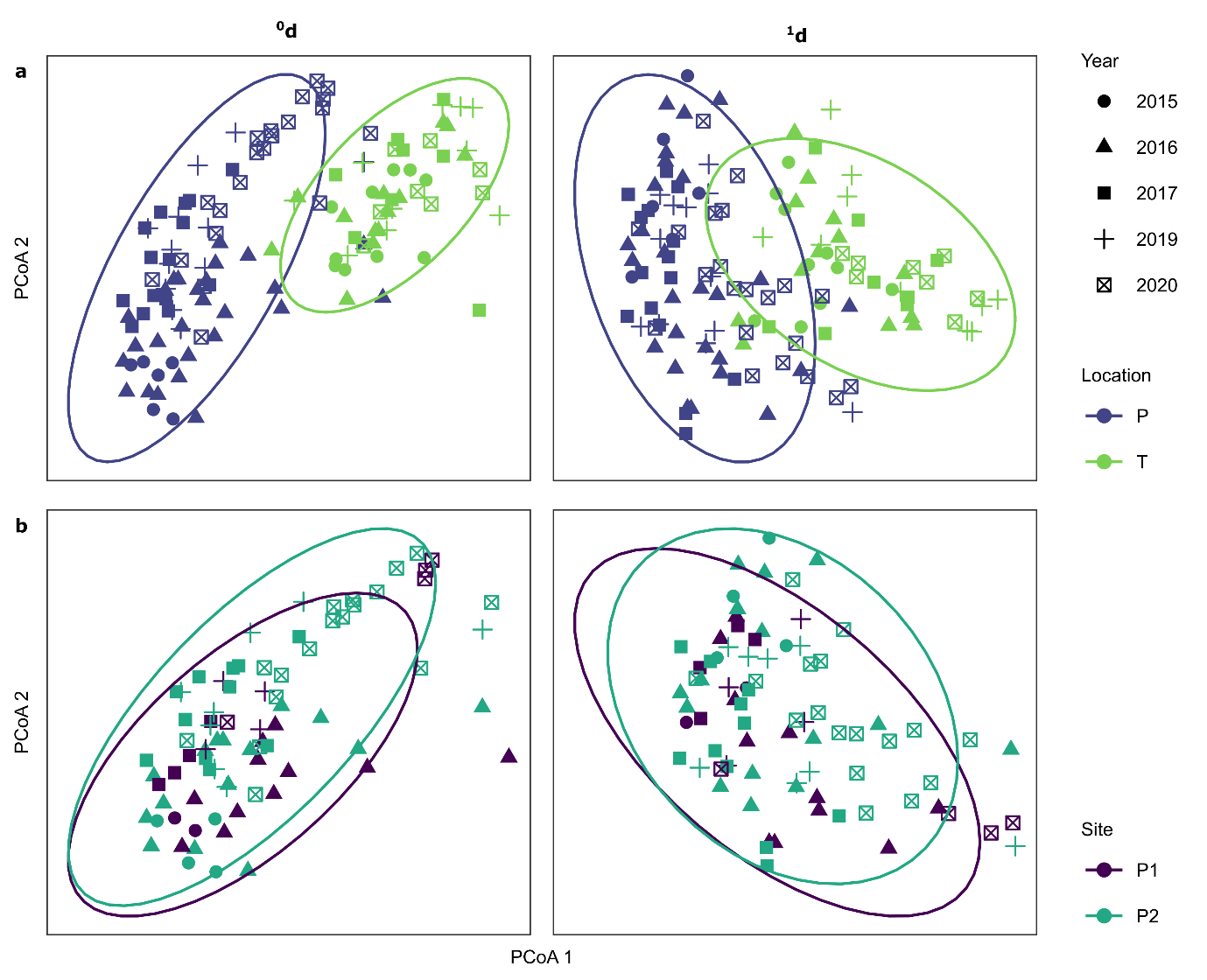


**Figure S9.** The top ones (panel a) are a comparison of location P an T. The bottom ones (pane b) is for location P, where the two subsites P1 and P2 are compared.


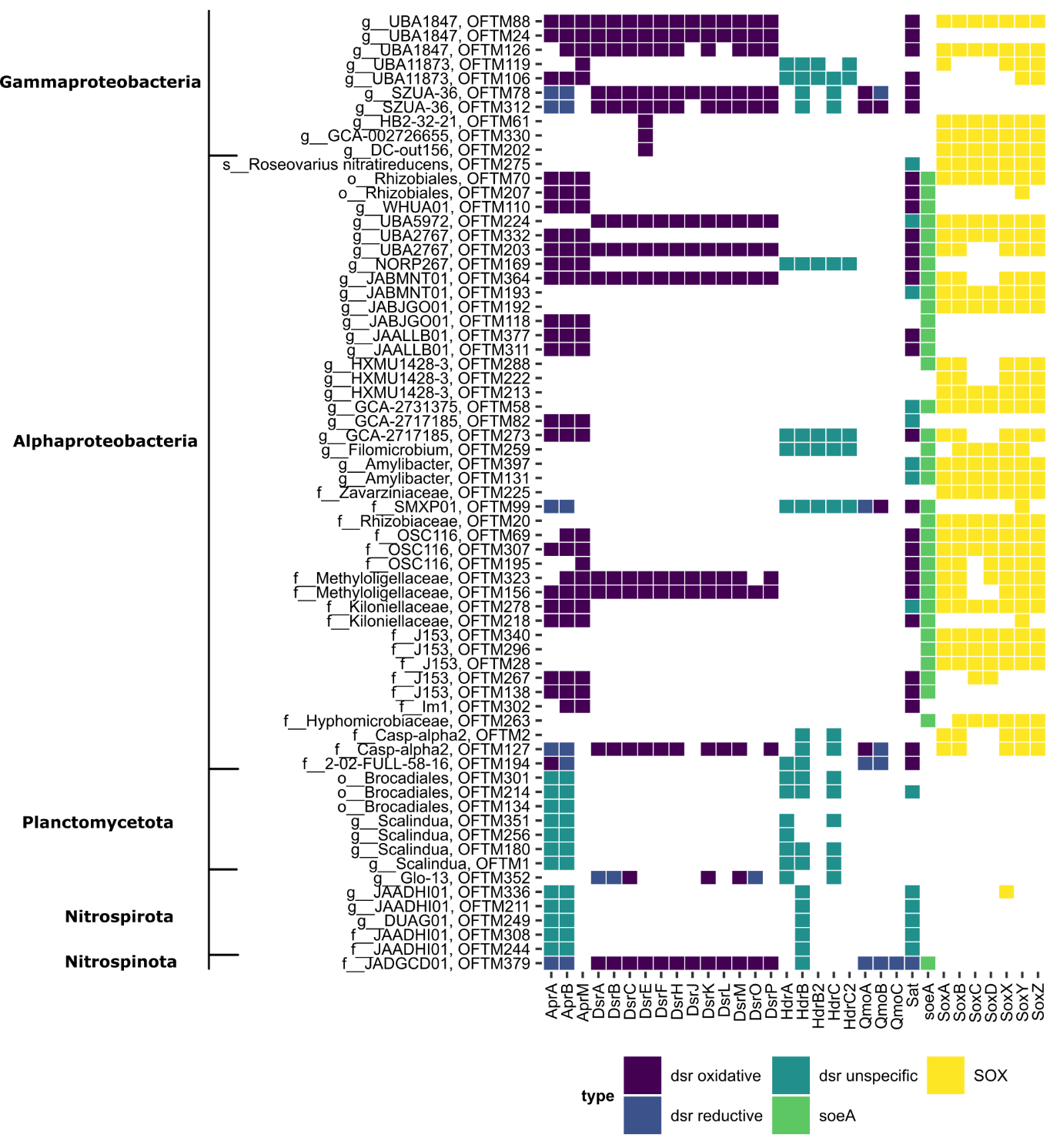


**Figure S10**. Analysis of Dsr and Sox genes. Only MAGs with AprAB or dsrAB are shown. Sox genes and soeA were annotated with DRAM. Apr, Dsr, Sat, Hdr and Qmo genes were annotated with DiSCo


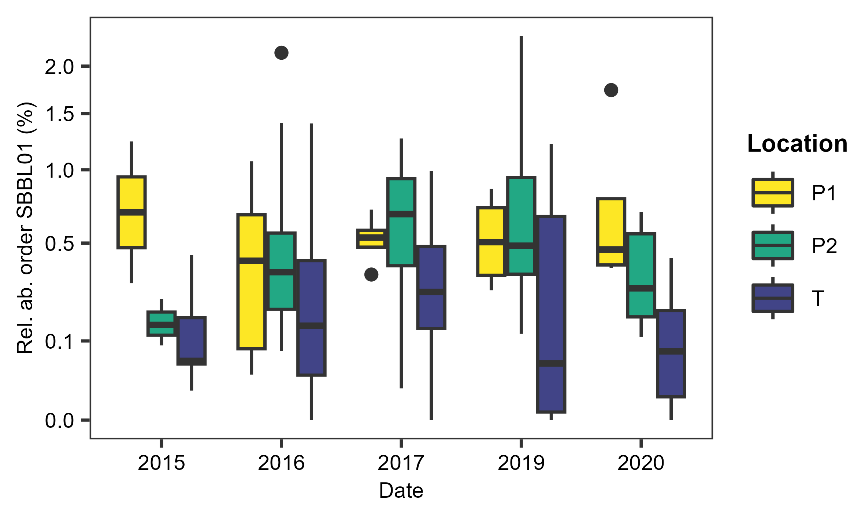


**Figure S11.** Relative abundance of the order *SBBL01* in the 16S dataset; each circle represents a sample. A square root transformation is used in the Y-axis to show differences at low relative abundances.


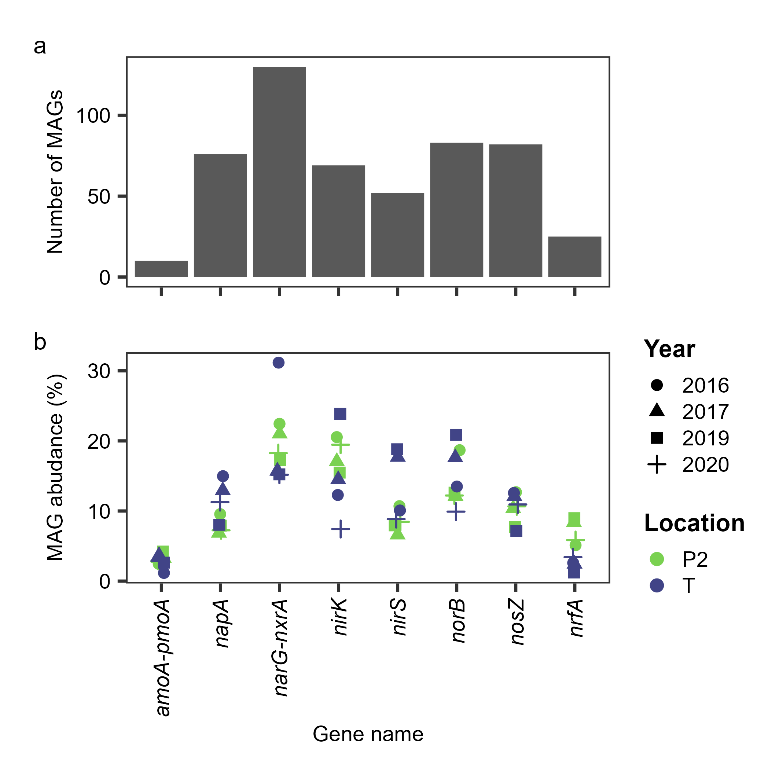


**Figure S12:** Potential for nitrogen cycling in microbial community members of Oslofjord tunnel biofilms. Number of MAGs with genes for nitrogen cycling (A); and relative abundance of MAGs with genes for nitrogen cycling (B).
